# Generative diffeomorphic modelling of large MRI data sets for probabilistic template construction

**DOI:** 10.1016/j.neuroimage.2017.10.060

**Published:** 2018-02-01

**Authors:** Claudia Blaiotta, Patrick Freund, M. Jorge Cardoso, John Ashburner

**Affiliations:** a Wellcome Trust Centre for Neuroimaging, University College London, London, UK; b Spinal Cord Injury Center Balgrist, University Hospital Zurich, University of Zurich, Zurich, Switzerland; c Translational Imaging Group, CMIC, University College London, London, UK

**Keywords:** Atlas construction, Image segmentation, Image registration, Generative modelling, MRI

## Abstract

In this paper we present a hierarchical generative model of medical image data, which can capture simultaneously the variability of both signal intensity and anatomical shapes across large populations. Such a model has a direct application for learning average-shaped probabilistic tissue templates in a fully automated manner. While in principle the generality of the proposed Bayesian approach makes it suitable to address a wide range of medical image computing problems, our work focuses primarily on neuroimaging applications. In particular we validate the proposed method on both real and synthetic brain MR scans including the cervical cord and demonstrate that it yields accurate alignment of brain and spinal cord structures, as compared to state-of-the-art tools for medical image registration. At the same time we illustrate how the resulting tissue probability maps can readily be used to segment, bias correct and spatially normalise unseen data, which are all crucial pre-processing steps for MR imaging studies.

## Highlights

• We present a generative modelling framework to process large MRI data sets.
• The proposed framework can serve to learn average-shaped tissue probability maps and empirical intensity priors.
• We explore semi-supervised learning and variational inference schemes.
• The method is validated against state-of-the-art tools using publicly available data.

## Introduction

Over the past two decades neuroimaging techniques have been widely recognised has powerful non-invasive tools to answer neuroscientific questions regarding, for instance, the variability of neuroanatomy across different populations (Good et al., 2001, Ashburner and Friston, 2000), the role of structural changes in determining disease onset and progression (Fox et al., 2000, Chetelat et al., 2005, Brex et al., 2002), as well as the relationships between neural activations and mental functions (Friston et al., 1996) during common cognitive, somatosensory or motor tasks.

One of the main challenges, which is encountered in all neuroimaging studies, originates from the difficulty of mapping between different anatomical shapes. In particular, a fundamental problem arises from having to ensure that this mapping operation preserves topological properties and that it provides, not only anatomical, but also functional overlap between distinct instances of the same anatomical object (Brett et al., 2002).

This explains the rapid development of the discipline known as computational anatomy (Grenander and Miller, 1998), which aims to provide mathematically sound tools and algorithmic solutions to model high-dimensional anatomical shapes, with the ultimate goal of encoding, or accounting for, their variability. In this framework, the problem of anatomical mapping is most commonly addressed by assuming that a population of subjects can be fully described by means of an average-shaped template, together with a set of high-dimensional transformations, which, once applied to the template, generate the observed anatomical configurations (Thompson et al., 2000). In this setting, an ideal template is one that can accurately be matched to the observed data while minimising the geodesic distance between the template itself and each observation (Avants and Gee, 2004).

One of the practical advantages of this approach is that, once a template has been constructed, it can be used to model unseen data drawn form the same population, thus providing an ideal reference coordinate system for neuroimaging studies and for statistical testing of neuroscientific hypotheses.

In this paper we propose a general modelling scheme and a training algorithm, which, given a large cross-sectional data set of MR scans, can learn a set of average-shaped tissue probability maps, either in an unsupervised or semi-supervised manner. This is achieved by building a hierarchical generative model of MR data, where image intensities are captured using multivariate Gaussian mixture models, after diffeomorphic warping (Ashburner and Friston, 2011, Joshi et al., 2004) of a set of unknown probabilistic templates, which act as anatomical priors. In addition, intensity inhomogeneity artefacts are explicitly represented in our model, meaning that the input data does not need to be bias corrected prior to model fitting.

Our work builds on a number of state-of-the-art techniques, some of which were already explored and validated individually as part of our previous work (Ashburner and Friston, 2005, Ashburner and Friston, 2011, Blaiotta et al., 2016). In particular, we rely on the variational Bayes expectation-maximisation (VBEM) algorithm for image segmentation proposed in Blaiotta et al. (2016), which is a robust generalisation of the segmentation method implemented in the SPM12 software (Ashburner and Friston, 2005). Additionally, we exploit the diffeomorphic image registration framework described in Ashburner and Friston (2011), which is an accurate, fast converging and memory efficient strategy to align complex anatomical shapes using diffeomorphic transformations.

To the best of our knowledge, the particular mathematical formulation that we adopt to combine such modelling techniques has never been adopted before. The resulting approach enables processing simultaneously a large number of MR scans in a groupwise fashion and particularly it allows the tasks of image segmentation, image registration, bias correction and atlas construction to be solved by optimising a single objective function, with one iterative algorithm. This is in contrast to a commonly adopted approach to mathematical modelling, which involves a pipeline of multiple model fitting strategies that solve sub-problems sequentially, without taking into account their circular dependencies. Our strategy instead, addresses the fact that learning anatomical priors requires collecting accurate segmentations from a training population, while such segmentations can only be obtained if suitable priors are available. Thus, the two modelling problems are solved more effectively within an integrated framework, rather than independently.

Related work was done by Bhatia et al. (2007), however their method relies on classical point estimation techniques to perform image segmentation, as opposed to our variational approach, which instead allows estimating full posteriors on the intensity distribution parameters. Additionally we incorporate bias correction and also explore semi-supervised learning, as opposed to the fully unsupervised scheme adopted in their work. The work presented in Lorenzen et al. (2004) is also along a similar line but it only address the problem of Bayesian diffeomorphic template construction given a set of pre-computed segmentations, without embedding image segmentation and atlas construction in a single mathematical model of the data.

The main aim of this paper is to demonstrate the methodological validity of the proposed approach, which, for this purpose, has been thoroughly tested on both synthetic and real MR neuroimaging data sets. However, the method is a general one and, since it was not tuned or optimised for a particular type of imaging data, we believe that it holds a potential for application in a wider range of medical image computing problems, some of which we aim to explore as part of our future work.

## Methods

Let us consider a population of *M* subjects belonging to a homogeneous group, from an anatomical point of view, and let us assume that *D* image volumes of different contrast are available for each subject.

From a generative perspective, the image intensities $X={\left\{X_{i}\right\}}_{i=1,\ldots ,M}$, which constitute the observed data, can be thought of as being generated by sampling from *D*-dimensional Gaussian mixture probability distributions, after non-linear warping of a probabilistic anatomical atlas (Evans et al., 1994). The use of Gaussian mixture models to capture the probability density function of MR data is a well established approach (Ashburner and Friston, 2005, Greenspan et al., 2006, Zhang et al., 2001). For an extensive review of such methods see Balafar (2014).

The probabilistic atlas carries prior anatomical knowledge, in the form of average shaped tissue probability maps. From a mathematical modelling point of view, the atlas encodes local mixing proportions ${\text{Θ}}_{\pi }={\left\{\pi_{j}\right\}}_{j=1,\ldots ,N_{\pi }}$ of the mixture model, with *j* being an index set over the $N_{\pi }$ template voxels (for a list of all the mathematical symbols used in this section see Table 1). Each vector $\pi_{j}$ has *K* elements, which indicate the prior probability of voxel *j* belonging to one of *K* tissue classes. These spatially varying mixing proportions can also be thought of as the coefficients used to parametrise *K* continuous scalar functions of space ${\left\{\pi_{k}\right\}}_{k=1,\ldots ,K}$.

**Table 1:** List of mathematical symbols used in this paper.

Symbol	Meaning
$x_{ij}$	Observed image intensity at voxel *j* for subject *i*.
$z_{ij}$	Vector of latent class membership probabilities.
$\pi_{j}$	Tissue priors at voxel *j*.
$\mu_{ik}$	Mean intensity of class *k* for subject *i*.
$\Sigma_{ik}$	Covariance of intensities for class *k* and subject *i*.
$W_{0k}$	Scale matrix of Wishart prior distribution on $\Lambda_{k}={\left(\Sigma_{k}\right)}^{-1}\text{.}$
$\nu_{0k}$	Degrees of freedom of Wishart prior distribution on $\Lambda_{k}$.
$m_{0k}$	Mean of Gaussian prior distribution over $\mu_{k}$
$\beta_{0k}$	Scaling hyperparameter of Gaussian prior distribution over $\mu_{k}$
$\alpha_{0}$	Hyperparameter governing the Dirichlet prior on π.
${\text{Θ}}_{\beta }$	Bias field parameters.
$\mu_{\beta }$	Prior mean of bias parameters.
$\Sigma_{\beta }$	Prior covariance matrix of bias parameters.
${\text{Θ}}_{a}$	Affine transformation parameters.
$\mu_{a}$	Prior mean of affine transformation parameters.
$\Sigma_{a}$	Prior covariance matrix of affine transformation parameters.
$w_{i}$	Weights for rescaling the tissue priors.
$u_{ij}$	Initial velocity at voxel *j* for subject *i*.
$L_{u}$	Differential operator to compute penalty on $u_{i}$.

### Tissue priors

Each image voxel, $j\in \left\{1,\ldots ,N_{i}\right\}$^1^ for each subject i∈{1,…,M} is considered as being drawn from *K* possible tissue classes. The following prior latent variable model defines the probability of finding tissue type *k*, at a specific location *j* (i.e. centre of voxel *j*), in image *i*, prior to observing the corresponding image intensity signal

(1) $$p\left(z_{ijk}=1|{\text{Θ}}_{\pi },{\text{Θ}}_{w},{\text{Θ}}_{u}\right)=\frac{w_{ik}\;\pi_{k}\left(\xi_{i}\left(y_{j}\right)\right)}{\sum_{c=1}^{K}w_{ic}\;\pi_{c}\left(\xi_{i}\left(y_{j}\right)\right)}\;,$$

or equivalently

(2) $$p\left(z_{ij}|{\text{Θ}}_{\pi },{\text{Θ}}_{w},{\text{Θ}}_{u}\right)=\prod_{k=1}^{K}{\left(\frac{w_{ik}\;\pi_{k}\left(\xi_{i}\left(y_{j}\right)\right)}{\sum_{c=1}^{K}w_{ic}\;\pi_{c}\left(\xi_{i}\left(y_{j}\right)\right)}\right)}^{z_{ijk}}\;\text{.}$$

Class memberships, for each subject and each voxel, are encoded in the latent variable $z_{ij}$, using a one-of-K scheme (i.e. $z_{ij}$ is *K*-dimensional vector with all elements equal to zero except for one, which is equal to one). In particular, $z_{ijk}\in \left\{0,1\right\}$ is equal to one if voxel *j* of image *i* belongs to tissue class *k* and zero otherwise, which also explains the equivalence of (1), (2). ${\left\{\pi_{k}\right\}}_{k=1,\ldots ,K}$ are continuous scalar functions $\pi_{k}:{\text{Ω}}_{\pi }\to \mathbb{R}$, defined on the template domain ${\text{Ω}}_{\pi }$. Such functions are common across the entire population, which satisfy the constraint

(3) $$\sum_{k=1}^{K}\pi_{k}\left(y\right)=1\;,\forall y\in {\text{Ω}}_{\pi }\subset {\mathbb{R}}^{3}\;,$$

with y being a continuous coordinate vector field. Global weights ${\text{Θ}}_{w}={\left\{w_{i}\right\}}_{i=1,\ldots ,M}$, with $w_{i}\in {\mathbb{R}}^{K}$, are introduced to further compensate for individual differences in tissue composition.

In equation (1), $\xi_{i}$ denotes a generic spatial transformation, parametrised by ${\text{Θ}}_{u}$, which allows projecting prior anatomical information onto individual data, with $\xi_{i}:{\text{Ω}}_{i}\to {\text{Ω}}_{\pi }$ being a continuous mapping from the domain ${\text{Ω}}_{i}\subset {\mathbb{R}}^{3}$ of image *i*, into the space of the tissue priors ${\text{Ω}}_{\pi }\subset {\mathbb{R}}^{3}$. In this work we adopt a large deformation diffeomorphic model (Ashburner and Friston, 2011, Beg et al., 2005, Trouvé, 1998), where, as explained in the following subsection, the diffeomorphisms are parametrised by means of an initial velocity field, denoted by u.

Since digital image data is a discrete signal, defined on a tridimensional voxel grid, each mapping $\xi_{i}$ needs to be discretised as well, via sampling at the centre of every voxel $j\in \left\{1,\ldots ,N_{i}\right\}$, to give the discrete mapping ${\left\{\xi_{i}\left(y_{j}\right)\right\}}_{j=1,\ldots ,N_{i}}$ that appears in (1).

As opposed to the modelling approach described in Ashburner and Friston (2005) and Blaiotta et al. (2016), where the tissue priors were considered as fixed and known *a priori* quantities, here the tissue probability maps are treated as random variables, whose point estimates or full posteriors can be inferred via model fitting (Bhatia et al., 2007, Ribbens et al., 2014).

For this purpose, a finite dimensional parametrisation of the continuous functions ${\left\{\pi_{k}\right\}}_{k=1,\ldots ,K}$ needs to be defined. Typically, whenever a continuous function needs to be reconstructed from a finite discrete sequence, it is possible to formulate the problem as an interpolation that makes use of a finite set of coefficients and continuous basis functions. Since the priors ${\left\{\pi_{k}\right\}}_{k=1,\ldots ,K}$ are bounded to take values in the interval [0,1] on the entire domain ${\text{Ω}}_{\pi }$ (see equation (3)), not all basis functions are well suited here. For this reason we use first degree B-splines, which ensure that the tissue priors satisfy the above mentioned constraint, while being quite a computationally efficient choice compared to higher order interpolation methods. The coefficients used to parametrise the tissue priors belong to the discrete set ${\text{Θ}}_{\pi }={\left\{\pi_{j}\right\}}_{j=1,\ldots ,N_{\pi }}$ of *K*-dimensional vectors, with

(4) $$\sum_{k=1}^{K}\pi_{jk}=1,\;\forall j\in \left\{1,\ldots ,N_{\pi }\right\}\;\text{.}$$

and $N_{\pi }$ being the number of template voxels. Such coefficients can be learned directly from the data, as it will be shown in the following section.

Additionally, prior distributions on the parameters ${\left\{\pi_{j}\right\}}_{j=1,\ldots ,N}$ can be introduced (Bishop, 2006). Dirichlet priors are the most convenient choice here, since they are conjugate to multinomial forms of the type in (2), and they can be expressed as

(5) $$p\left(\pi_{j}\right)=\text{Dir}\left(\pi_{j}|\alpha_{0}\right)=C\left(\alpha_{0}\right)\prod_{k=1}^{K}\pi_{jk}^{\alpha_{k}-1}\;,$$

where the normalising constant is given by

(6) $$C\left(\alpha_{0}\right)=\frac{\text{Γ}\left(\bar{\alpha }\right)}{\text{Γ}\left(\alpha_{1}\right)\ldots \text{Γ}\left(\alpha_{k}\right)}\;,$$

with Γ(⋅) being the gamma function and

(7) $$\bar{\alpha }=\sum_{k=1}^{K}\alpha_{k}\;\text{.}$$

### Diffeomorphic image registration

As anticipated in the previous sections, the generative interpretation of imaging data that this work relies on involves warping an unknown, average-shaped atlas to match a series of individual scans.

Such a problem, that is to say template matching via non-rigid registration, has been largely explored in medical imaging, mainly for solving image segmentation or structural labeling problems, in an automated fashion (Ashburner and Friston, 2005, Shen and Davatzikos, 2004, Christensen, 1999, Chui et al., 2001, Bajcsy et al., 1983, Iglesias et al., 2012, Pluta et al., 2009, Warfield et al., 1999, Khan et al., 2008, Bowden et al., 1998).

Indeed, the modelling of spatial mappings between different anatomies can be approached in a variety of manners, depending on the adopted model of shape and on the objective function (i.e. similarity metric and regularisation) that the optimisation is based on, thus leading to a variety of algorithms with remarkably different properties (Penney et al., 1998, Denton et al., 1999, Klein et al., 2009).

The work presented here is formulated according to the Large Deformation Diffeomorphic Metric Mapping (LDDMM) framework (Younes, 2010), where the transformations mapping between the source images and the target image are assumed to belong to a Riemannian manifold 2 of diffeomorphisms (Ashburner, 2007). A diffeomorphism ϕ:Ω→Ω is a smooth differentiable map (with a smooth differentiable inverse $\phi^{-1}$) defined on a compact, simply connected domain $\text{Ω}\subset {\mathbb{R}}^{3}$.

One way of constructing transformations belonging to the diffeomorphic group Diff(Ω) is to solve the following non-stationary transport equation (Joshi and Miller, 2000)

(8) $$\frac{\text{d}}{\text{d}t}\phi \left(y,t\right)=u\left(\phi \left(y,t\right),t\right),\;\phi \left(y,0\right)=y,\;t\in \left[0,1\right]\;,$$

where u(ϕ(y,t),t)∈ℋ is a time dependent, smooth velocity vector field, in the Hilbert space^3^ ℋ.

The initial map, at t=0, is equal to the identity transform ϕ(y,0)=y, while the final map, endpoint of the flow of the velocity field u, can be computed by integration on the unitary time interval t∈[0,1] (Beg et al., 2005).

(12) $$\phi \left(y,1\right)=\int_{0}^{1}u\left(\phi \left(y,t\right),t\right)\text{d}t+\phi \left(y,0\right)\;\text{.}$$

A procedure known as geodesic shooting (Miller et al., 2006, Ashburner and Friston, 2011, Allassonnière et al., 2005, Vialard et al., 2012, Beg and Khan, 2006) is applied, within the work presented here, to compute the final diffeomorphism given the initial map (i.e. the identity transform) and the velocity field at t=0. Such a procedure exploits the principle of conservation of momentum (Younes et al., 2009), which is given by $L_{u}^{†}L_{u}u_{t}$, with $L_{u}^{†}$ being the adjoint of the differential operator $L_{u}$, to integrate the dynamical system governed by (8) without having to compute and store an entire time series of velocity fields. The implementation adopted here relies on the work presented in Ashburner and Friston (2011).

A diffeomorphic path ϕ is not only differentiable, but also guaranteed to be a one-to-one mapping. Such a quality is highly desirable for finding morphological and functional correspondences between different anatomies without introducing tears or foldings, which would violate the conditions for topology preservation (Christensen, 1999). Additionally, the diffeomorphic framework provides metrics to quantitatively evaluate distances between anatomies or shapes. It should also be noted that diffeomorphisms are locally analogous to affine transformations (Avants et al., 2006).

In practice, finding an optimal diffeomorphic transformation to equation a pair, or a group, of images involves optimising an objective function (e.g. minimising a cost function), in the space ℋ of smooth velocity vector fields defined on the domain Ω. The required smoothness is enforced by constructing the norm on the space ℋ through a differential operator $L_{u}$ (Beg et al., 2005), such that a quantitative measure of smoothness can be obtained via

(13) $$ℛ\left(u\right)=||L_{u}u|{|}_{L^{2}}^{2}\;,$$

where u is a discretised version of u.

The form of the cost function will depend on how the observed data is modelled. For the work presented here, groupwise equationment is achieved via maximisation of the following variational objective function

(14) $$\begin{array}{l} E\left({\text{Θ}}_{u}\right)=E_{Z}\left[\text{log}p\left(\bar{Z}|{\text{Θ}}_{\pi },{\text{Θ}}_{w},{\text{Θ}}_{u}\right)\right]+\text{log}p\left({\text{Θ}}_{u}\right)+\text{const}\\ =\sum_{i=1}^{M}\sum_{j=1}^{N_{i}}\sum_{k=1}^{K}\gamma_{ijk}\text{log}\left(\frac{w_{ik}\pi_{k}\left(\phi_{i}\left(y_{j}\right)\right)}{\sum_{c=1}^{K}w_{ic}\;\pi_{c}\left(\phi_{i}\left(y_{j}\right)\right)}\right)\\ -\frac{1}{2}\sum_{i=1}^{M}||L_{u}u_{i}|{|}_{L^{2}}^{2}+\text{const}\;, \end{array}$$

where $Z={\left\{Z_{i}\right\}}_{i=1,\ldots ,M}$ is the set of latent variables across the entire population, ${\left\{\gamma_{ij}\right\}}_{i,j}={\{E\left[z_{ij}\right]\}}_{i,j}$ are *K*-dimensional vectors encoding the posterior probabilities of each voxel belonging to the *K* tissue types. The coordinate mappings ${\left\{\phi_{i}\right\}}_{i=1,\ldots ,M}$ are encoded in the parameter set ${\text{Θ}}_{u}$, which consists of *M* vectors of coefficients ${\left\{u_{i}\right\}}_{i=1,\ldots ,M}$, containing $3\times N_{i}$ elements each. Such coefficients can be used to construct continuous initial velocity fields via trilinear, or higher order, interpolation.

The posterior membership probabilities ${\left\{\gamma_{ij}\right\}}_{i,j}$ that appear in (14) can be computed by combining the prior latent variable model introduced in 2.1 with a class conditional likelihood model of image intensities, which will be described in subsection. In such a case, learning posterior label probabilities can be addressed as a standard mixture distribution inference problem, which can be conveniently solved using the expectation-maximisation algorithm or its variational extensions (Bishop, 2006, Blaiotta et al., 2016), thus leading to a fully unsupervised learning scheme.

Alternatively, when manual labels are available, binary posterior class probabilities can be derived directly from such categorical annotations, without performing inference from the observed image intensity data. In particular, if all input data has been manually labelled, then the resulting algorithm would implement a fully supervised learning strategy. Instead, if only some of the data has associated training labels, a hybrid approach can be adopted, which would fall into the category of semisupervised learning (Chapelle et al., 2006, Filipovych et al., 2011).

Essentially these three approaches, namely unsupervised, semisupervised and fully supervised, differ in the relative ratio between the number of labelled and unlabelled voxels. Even so, they can all be framed within an expectation-maximisation setting, where depending on whether a voxel is labelled or not, the E-step is performed differently. Specifically, if $x_{ij}$ is an unlabelled observation

(15) $$\begin{array}{l} \gamma_{ijk}=p\left(z_{ijk}=1|x_{ij},\text{Θ}\right)\\ =\frac{p\left(x_{ij}|z_{ijk}=1,\text{Θ}\right)p\left(z_{ijk}=1|\text{Θ}\right)}{\sum_{c=1}^{K}p\left(x_{ij}|z_{ijc}=1,\text{Θ}\right)p\left(z_{ijc}=1|\text{Θ}\right)}\;, \end{array}$$

with Θ being the current estimate of model parameters at each iteration of the algorithm. Instead, if $x_{ij}$ has been manually labelled

(16) $$\gamma_{ijk}=p\left(z_{ijk}=1|l_{ij}\right)=\{\begin{array}{cc} 1, & \text{if }l_{ij}=k\\ 0, & \text{if }l_{ij}\neq k \end{array}$$

where $l_{ij}$ is a categorical manual label assigned to voxel *j* in image *i*.

Finally, it is also possible to take into account the uncertainty inherent in the process of manual rating. In such a case, the actual posterior probabilities, for labelled observations, can be computed by making use of the categorical output of manual labelling together with an estimate of the rater sensitivity and with a generative intensity model.

Making use of Bayes rule, this gives

(17) $$\begin{array}{l} \gamma_{ijk}=p\left(z_{ijk}=1|x_{ij},\text{Θ},l_{ij}\right)\\ =\frac{p\left(x_{ij}|z_{ijk}=1,\text{Θ}\right)p\left(z_{ijk}=1|\text{Θ}\right)p\left(z_{ijk}=1|l_{ij}\right)}{\sum_{c=1}^{K}p\left(x_{ij}|z_{ijc}=1,\text{Θ}\right)p\left(z_{ijc}=1|\text{Θ}\right)p\left(z_{ijc}=1|l_{ij}\right)}\;, \end{array}$$

where $p\left(z_{ijk}=1|l_{ij}\right)$ indicates the probability of voxel *j* in image *i* belonging to class *k*, given the manual label attributed to the same voxel.

A simple model for this, is

(18) $$p\left(z_{ijk}=1|l_{ij}\right)=\{\begin{array}{cc} \zeta_{l}, & \text{if }l_{ij}=k\\ \frac{1-\zeta_{l}}{K-1}, & \text{if }l_{ij}\neq k \end{array}$$

where $\zeta_{l}$ is the sensitivity of the rater that generated the set of labels ${\left\{l_{ij}\right\}}_{j=1,\ldots ,N}$ for image *i*. The problem of how to evaluate the performance of a manual or automated rater is not addressed here. For instance, a probabilistic scheme, which has been widely used to assess segmentation performance in medical imaging, is presented in Warfield et al. (2004).

In the remainder of this paper we will be focusing on the semi-supervised approach and we will consider the general case where only some voxels in few images have been manually labelled by adopting the model in (17) with a fixed sensitivity parameter $\zeta_{l}$ equal to 0.9.

### Combining diffeomorphic with affine registration

Anatomical shapes are very high dimensional objects. The diffeomorphic model described in the previous section, which is encoded using $3\times N_{i}$ free parameters, can account for a significant amount of shape variability in the observed data. Nevertheless, it is still convenient, mainly for computational reasons, to combine such a local, high dimensional shape model with global, lower dimensional transformations, such as rigid body or affine transforms. In fact, by beginning to solve the registration problem from the coarsest deformation components (e.g. rigid body or affine), it is possible to ensure that the subsequent diffeomorphic registration starts from a good initial estimate of image alignment, that is to say closer to the desired global optimum (Lester and Arridge, 1999). This makes the optimisation problem faster to solve and at the same time it reduces significantly the chance of registration failure (Modersitzki, 2004). It is relatively common for non-linear registration algorithms to perform poorly in the presence of a large translational or size mismatch between the reference and the target images (Jenkinson and Smith, 2001).

A possible parametrisation that combines affine and diffeomorphic transformations is

(19) $$\xi_{i}\left(y\right)=T_{i}\;\phi_{i}\left(y\right)+t_{i},\;\;\forall y\in {\text{Ω}}_{i}\;,$$

where $\xi_{i}\left(y\right)$ is the resulting mapping from image of subject *i* into the template space. Such a mapping is obtained by affine transforming the diffeomorphic deformation field $\phi_{i}$. The transformation matrix $T_{i}$ encodes nine degrees of freedom (rotation, zooming and shearing) and is computed via an exponential map $T_{i}=\text{exp}\left(Q_{i}\left(a_{i}\right)\right)$ with $Q_{i}\left(a_{i}\right)\in ga\left(3\right)$, where ga(3) is the Lie algebra for the affine group in three dimension GA(3) and $a_{i}$ is a vector of nine parameters (Ashburner and Ridgway, 2013). Translations are modelled by the vector $t_{i}\in {\mathbb{R}}^{3}$. The entire set of affine parameters is denoted as ${\text{Θ}}_{a}={\left\{a_{i},t_{i}\right\}}_{i=1,\ldots ,M}$.

### Intensity model

From a general probabilistic perspective, classification of tissue types based on MR signal intensities requires a model of the observed data that is capable of capturing the probability of occurrence of each signal sample value $x_{ij}$, provided that the true labels are known. In other words, the problem breaks down into defining suitable conditional probabilities $p\left(x_{ij}|z_{ijk}=1\right)$, for each k={1,…,K} and then applying Bayes rule to infer the posterior class probabilities.

In the model adopted here, image intensity distributions are represented as Gaussian mixtures, with the unknown mean $\mu_{ik}$ and covariance matrix $\Sigma_{ik}$ of each Gaussian component *k*, for subject *i*, being governed by Gaussian-Wishart priors (Bishop, 2006, Blaiotta et al., 2016).

Correction of intensity inhomogeneities is also performed within the same modelling framework and it involves multiplying the uncorrected intensities of each image volume by a bias field, which is modelled as the exponential of a weighted sum of discrete cosine transform basis functions (Styner et al., 2000, Ashburner and Friston, 2005). Such an approach is conceptually equivalent to scaling the probability distributions of all Gaussian components by a local scale parameter, which is the bias itself, such that

(20) $$p\left(x_{ij}|z_{ijk},\mu_{ik},\Sigma_{ik},{\text{Θ}}_{\beta }\right)=N\left(x_{ij}|{\hat{\mu }}_{ik},{\hat{\Sigma }}_{ik}\right)\;,$$

with

(21) $$\begin{array}{l} {\hat{\mu }}_{ik}={\left(\text{diag}\left(b_{ij}\right)\right)}^{-1}\mu_{ik}\;,\\ {\hat{\Sigma }}_{ik}={\left(\text{diag}\left(b_{ij}\right)\right)}^{-1}\Sigma_{ik}{\left(\text{diag}\left(b_{ij}\right)\right)}^{-1}\;, \end{array}$$

where N indicates a Normal distribution, ${\text{Θ}}_{\beta }$ denotes the set of bias field parameters and $b_{ij}$ is a *D*-dimensional vector representing the bias at voxel *j* of subject *i* for each of the *D* imaging modalities. The result in (20) is independent from the particular parametrisation of $b_{ij}$, as long as the bias is multiplicative, and it can be easily proven by applying the change of variable $x_{ij}=c_{ij}/b_{ij}$, with $c_{ij}$ being the corrected image intensity

(22) $$\begin{array}{l} p\left(x_{ij}|z_{ijk},\mu_{ik},\Sigma_{ik},{\text{Θ}}_{\beta }\right)=\\ \text{det}\left(\text{diag}\left(b_{ij}\right)\right)p\left(c_{ij}|z_{ijk},\mu_{ik},\Sigma_{ik},{\text{Θ}}_{\beta }\right)=\\ \text{det}\left(\text{diag}\left(b_{ij}\right)\right)N\left(c_{ij}|\mu_{ik},\Sigma_{ik}\right)=N\left(x_{ij}|{\hat{\mu }}_{ik},{\hat{\Sigma }}_{ik}\right)\;\text{.} \end{array}$$

### Graphical model

**Fig. 1:**
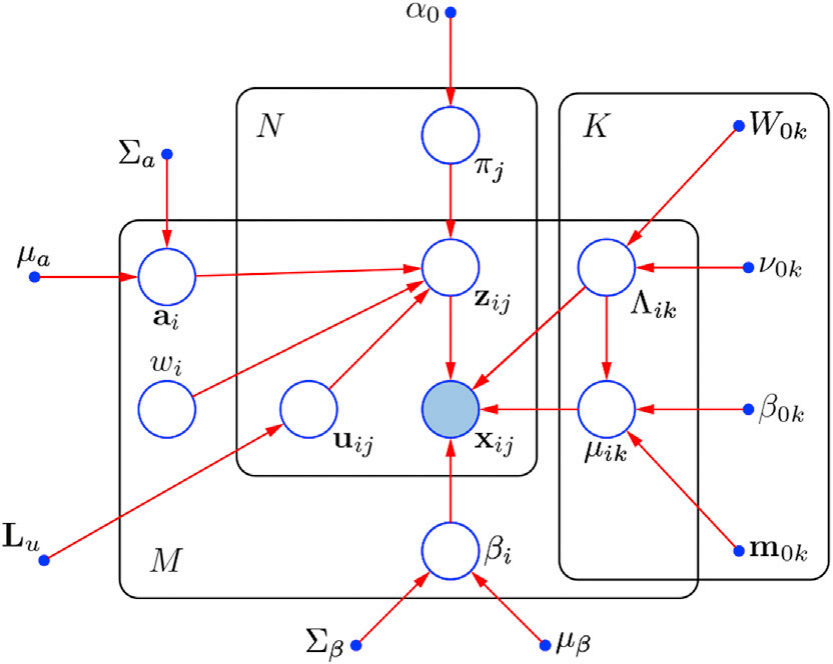
Graphical representation of the model adopted in this paper. Observed variables $\left\{x_{ij}\right\}$ are represented by a filled circle. Latent variables $\left\{z_{ij}\right\}$ as well as model parameters are depicted as unfilled circles. Blue solid dots correspond to fixed hyperparameters. The so called plate notation is adopted to indicated repeated variables. Symbols referring to all variables and parameters are listed in Table 1.

A graphical representation of the model adopted in this paper is depicted in Fig. 1, while a legend of the symbols used to indicate the different variables can be found in Table 1.

Given such a model, it is possible to define the following variational objective function ℒ, which constitutes a lower bound on the logarithm of the marginal joint probability $p\left(\bar{X},{\text{Θ}}_{\beta },{\text{Θ}}_{a},{\text{Θ}}_{u},{\text{Θ}}_{\pi }|{\text{Θ}}_{w}\right)$, such that

(23) $$\text{log}p\left(\bar{X},{\text{Θ}}_{\beta },{\text{Θ}}_{a},{\text{Θ}}_{u},{\text{Θ}}_{\pi }|{\text{Θ}}_{w}\right)\geq ℒ$$

and

(24) $$ℒ=E_{Z,{\text{Θ}}_{\mu },{\text{Θ}}_{\text{Σ}}}\left[\text{log}p\left(X|Z,{\text{Θ}}_{\mu },{\text{Θ}}_{\text{Σ}},{\text{Θ}}_{\beta }\right)\right]+E_{Z}\left[\text{log}p\left(Z|{\text{Θ}}_{\pi },{\text{Θ}}_{w},{\text{Θ}}_{u},{\text{Θ}}_{a}\right)\right]+E_{{\text{Θ}}_{\mu },{\text{Θ}}_{\text{Σ}}}\left[\text{log}p\left({\text{Θ}}_{\mu },{\text{Θ}}_{\text{Σ}}\right)\right]+\text{log}p\left({\text{Θ}}_{\pi }\right)+\text{log}p\left({\text{Θ}}_{\beta }\right)+\text{log}p\left({\text{Θ}}_{a}\right)+\text{log}p\left({\text{Θ}}_{u}\right)-E_{Z}\left[\text{log}q\left(Z\right)\right]-E_{{\text{Θ}}_{\mu },{\text{Θ}}_{\text{Σ}}}\left[\text{log}q\left({\text{Θ}}_{\mu },{\text{Θ}}_{\text{Σ}}\right)\right]\;,$$

where the expectations indicated as $E_{Z}$ and $E_{{\text{Θ}}_{\mu },{\text{Θ}}_{\text{Σ}}}$ are computed with respect to variational posterior distributions (Blaiotta et al., 2016, Bishop, 2006), denoted by q(⋅), on the latent variables $\bar{Z}$ and on the Gaussian means and covariances $\left\{{\text{Θ}}_{\mu },{\text{Θ}}_{\text{Σ}}\right\}$, respectively. Optimisation of ℒ, which provides optimal parameter and hyperparameter estimates, will be discussed in the following section.

### Model fitting

The model described in the previous section can be fit to data sets of MR images by combining a variational expectation-maximisation (VBEM) algorithm with gradient based numerical optimisation techniques.

Indeed, the VBEM algorithm described in Blaiotta et al. (2016) is well-suited for addressing the model estimation problem discussed here, since it allows learning posterior distributions on the Gaussian mixture parameters, under the assumption that $q\left(\bar{Z},{\text{Θ}}_{\mu },{\text{Θ}}_{\text{Σ}}\right)$ factorises as $q\left(\bar{Z}\right)q\left({\text{Θ}}_{\mu },{\text{Θ}}_{\text{Σ}}\right)$ (Bishop, 2006), and at the same time it is able to transfer the information encoded in such posteriors by estimating empirical intensity priors for each tissue type. As shown in our previous work (Blaiotta et al., 2016), this approach has several advantages over maximum likelihood estimation, including lower vulnerability to overfitting, faster convergence and higher robustness against misregistration, which inevitably occurs in the early iterations of any image registration algorithm.

Additionally, the algorithm proposed in this paper loops over all subjects in the population and, for each subject, it iterates over estimating the Gaussian-Wishart posteriors, the bias field parameters, the affine parameters and the initial velocities, which are all treated as conditional optimisations. Subsequently the tissue probability maps and the intensity priors are updated and the whole cycle is repeated until convergence.

Estimation of the bias field parameters ${\text{Θ}}_{\beta }$ can be conveniently performed via non-linear optimisation techniques. Here the problem is solved using the Gauss-Newton method (Bertsekas, 1999) with a backtracking line search, so as to maximise the objective function in (24) with respect to ${\text{Θ}}_{\beta }$. The resulting implementation is very similar to the one described in Ashburner and Friston (2005), therefore further details are omitted here. Optimisation of the affine parameters ${\text{Θ}}_{a}={\left\{a_{i},t_{i}\right\}}_{i=1,\ldots ,M}$ can also be carried out by means of a Gauss-Newton scheme and a brief description of the required computations can be found Appendix A. For the update of the weight parameters ${\text{Θ}}_{w}$ we adopt the same strategy outlined in Ashburner and Friston, 2005, Blaiotta et al., 2016.

The following sections instead will present in detail the algorithmic scheme used to learn the average shaped tissue templates ${\text{Θ}}_{\pi }={\left\{\pi_{j}\right\}}_{j=1,\ldots ,N_{\pi }}$ and to estimate the set of initial velocity fields ${\text{Θ}}_{u}={\left\{u_{i}\right\}}_{i=1,\ldots ,M}$.

The pseudocode reported in alg:opt illustrates the different optimisation stages of the proposed algorithm. Essentially, the purpose of each stage within the inner loop is to optimise a subset of parameters for a particular subject, while keeping all the other parameters fixed at their current estimates. This strategy leverages the conditional dependencies among different variables in the model (such conditional dependencies are illustrated in Fig. 1), with the aim of learning more accurate parameters compared to those estimated by solving the different optimisation problems independently (Mahapatra and Sun, 2012, Ashburner and Friston, 2005, Meng and Rubin, 1993). The outer loop serves to regenerate group priors both in the form of tissue probability maps and parametric intensity priors.

Algorithm 1 Optimisation algorithm for generating average-shaped tissue probability maps using the approach presented in this paper.

**Figure:**
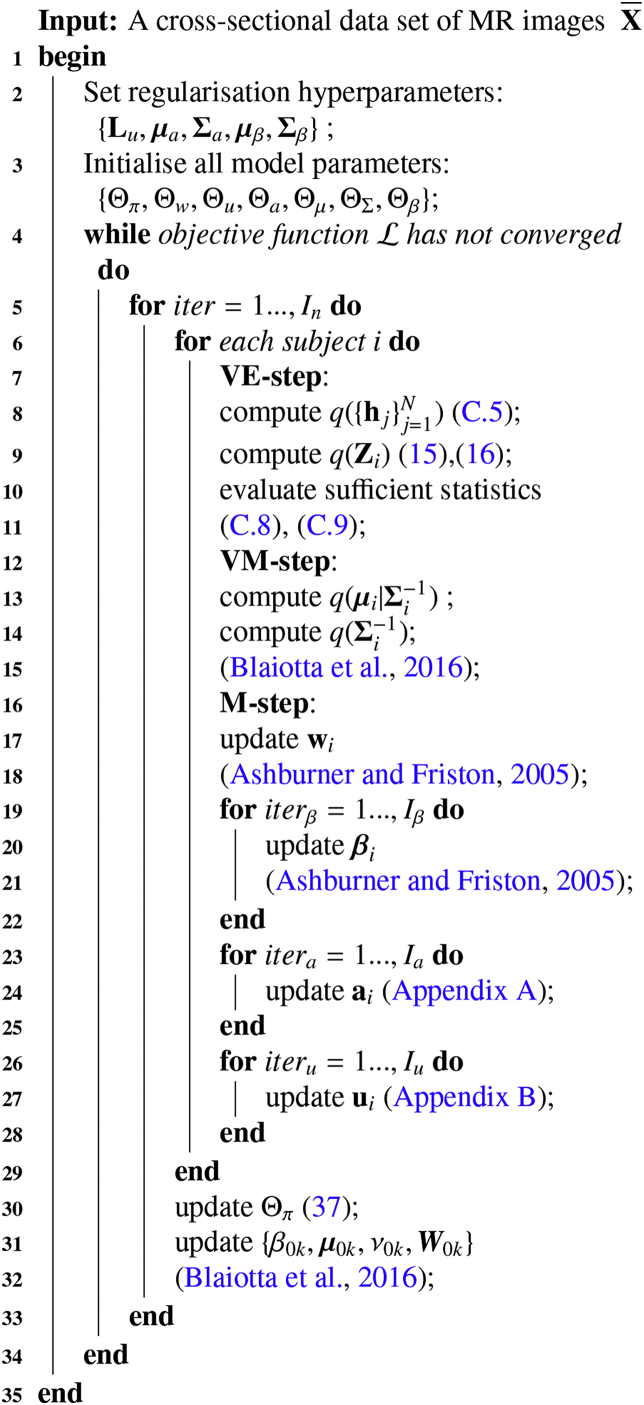

#### Updating the tissue priors

At each outer iteration of the algorithm, the tissue priors ${\text{Θ}}_{\pi }={\left\{\pi_{j}\right\}}_{j=1,\ldots ,N_{\pi }}$ need to be updated, given the current estimates of all the other parameters, which are kept fixed for each individual in the population.

Considering only the terms in (24) that depend on ${\text{Θ}}_{\pi }$ gives the following objective function, which has to be maximised with respect to ${\text{Θ}}_{\pi }$

(25) $$\begin{array}{l} {ℒ}_{\pi }=E_{Z}\left[\text{log}p\left(Z|{\text{Θ}}_{\pi },{\text{Θ}}_{w},{\text{Θ}}_{u},{\text{Θ}}_{a}\right)\right]+\text{log}p\left({\text{Θ}}_{\pi }\right)+\text{const}\\ =\sum_{i=1}^{M}\sum_{k=1}^{K}\int_{{\text{Ω}}_{i}}\gamma_{ik}\left(y\right)\text{log}\left(\frac{w_{ik}\pi_{k}\left(\xi_{i}\left(y\right)\right)}{\sum_{c=1}^{K}w_{ic}\;\pi_{c}\left(\xi_{i}\left(y\right)\right)}\right)\text{d}y\\ +\text{log}p\left({\text{Θ}}_{\pi }\right)+\text{const}\;\text{.} \end{array}$$

It should be noted that the parameters ${\text{Θ}}_{\pi }$ that need to be estimated are defined on the domain of the template ${\text{Ω}}_{\pi }$, rather than on the individual spaces ${\left\{{\text{Ω}}_{i}\right\}}_{i=1,\ldots ,M}$. For this reason equation (25), which is a sum of integrals on the native domains, needs to be mapped to ${\text{Ω}}_{\pi }$, by inverting the warps ${\left\{\xi_{i}\right\}}_{i=1,\ldots ,M}$, to give

(26) $$\begin{array}{l} {ℒ}_{\pi }^{\prime }=\sum_{i=1}^{M}\sum_{k=1}^{K}\int_{{\text{Ω}}_{\pi }}\text{det}\left(\frac{\partial \xi_{i}^{-1}}{\partial y}\right)\gamma_{ik}\left(\xi_{i}^{-1}\left(y\right)\right)\text{log}\left(\frac{w_{ik}\pi_{k}\left(y\right)}{\sum_{c}^{K}w_{ic}\;\pi_{c}\left(y\right)}\right)\text{d}y\\ +\text{log}p\left({\text{Θ}}_{\pi }\right)+\text{const}\;, \end{array}$$

where $\xi_{i}^{-1}$ is the transformation mapping from image *i* into the template space and the determinants of the Jacobian matrices of the deformations are included to preserve volumes after the change of variables.

Finally equation (26) is discretised on a regular voxel grid, whose centres have coordinates ${\left\{y_{j}\right\}}_{j=1,\ldots ,N_{\pi }}$, to give

(27) $$\begin{array}{l} {ℒ}_{\pi }^{\prime }=\sum_{i=1}^{M}\sum_{j=1}^{N_{\pi }}\sum_{k=1}^{K}\text{det}\left(J_{ij}^{\xi^{-1}}\right)\;\gamma_{ik}\left(\xi_{ij}^{-1}\right)\;\text{log}\left(\frac{w_{ik}\pi_{jk}}{\sum_{c=1}^{K}w_{ic}\;\pi_{jc}}\right)\\ +\text{log}p\left({\text{Θ}}_{\pi }\right)+\text{const}\;, \end{array}$$

where

(28) $$\xi_{ij}^{-1}=\xi_{i}^{-1}\left(y\right){|}_{y=y_{j}}\;,$$

(29) $$\text{det}\left(J_{ij}^{\xi^{-1}}\right)=\text{det}\left(\frac{\partial \xi_{i}^{-1}\left(y\right)}{\partial y}\right){|}_{y=y_{j}}\;,$$

(30) $$\pi_{jk}=\pi_{k}\left(y\right)|$$

The prior term $p\left({\text{Θ}}_{\pi }\right)$ is given by the following Dirichlet distribution

(31) $$p\left({\text{Θ}}_{\pi }\right)=\prod_{j=1}^{N_{\pi }}\text{Dir}\left(\pi_{j}|\alpha_{0}\right)=C\left(\alpha_{0}\right)\prod_{j=1}^{N_{\pi }}\prod_{k=1}^{K}\pi_{jk}^{\alpha_{0k}-1}\;\text{.}$$

Maximising equation (27) is a constrained optimisation problem, subject to

(32) $$\sum_{k=1}^{K}\pi_{jk}=1\;,\;\forall j\in \left\{1,\ldots ,N_{\pi }\right\}$$

A closed form solution could be easily found if the rescaling weights ${\left\{w_{i}\right\}}_{i=1,\ldots ,M}$ were all equal to one. In such a case

(33) $$\begin{array}{l} {ℒ}_{\pi }^{\prime }=\sum_{i=1}^{M}\sum_{j=1}^{N_{\pi }}\sum_{k=1}^{K}\text{det}\left(J_{ij}\right)\;\gamma_{ik}\left(\xi_{ij}^{-1}\right)\;\text{log}\left(\pi_{jk}\right)\\ +\sum_{j=1}^{N_{\pi }}\sum_{k=1}^{K}\left(\alpha_{0k}-1\right)\text{log}p\left(\pi_{jk}\right)+\text{const}\;, \end{array}$$

which could be maximised under the constraint (32), by making use of Lagrange multipliers (Falk, 1967), to give

(34) $$\pi_{jk}=\frac{N_{jk}+\alpha_{0k}-1}{\sum_{k=1}^{K}\left(N_{jk}+\alpha_{0k}\right)-K}\;,$$

with $N_{jk}=\sum_{i=1}^{M}\text{det}\left(J_{ij}\right)\;\gamma_{ik}\left(\xi_{ij}^{-1}\right)$.

This solution would provide maximum a posteriori point estimates of ${\text{Θ}}_{\pi }={\left\{\pi_{j}\right\}}_{j=1,\ldots ,N_{\pi }}$. However for this problem, it would also be possible to derive a full variational posterior distribution, which, like its prior, would take a Dirichlet form, with parameters $\alpha_{j}=\alpha_{0}+N_{j}$ .

When rescaling of the tissue priors is allowed the optimisation problem becomes more complex. The strategy adopted here consists in finding an approximate solution to the unconstrained optimisation problem by setting the derivatives of the objective function in (26) to zero

(35) $$\frac{\alpha_{0k}-1}{\pi_{jk}}+\sum_{i=1}^{M}\text{det}\left(J_{ij}^{\xi^{-1}}\right)\;\gamma_{ik}\left(\xi_{ij}^{-1}\right)\left(\frac{1}{\pi_{jk}}-\frac{w_{ik}}{\sum_{c=1}^{K}w_{ic}\pi_{jc}}\right)=0\;\text{.}$$

Solving with respect to $\pi_{jk}$, under the simplifying assumption that the term $\sum_{c=1}^{K}w_{ic}\pi_{jc}$ can be treated as a constant, gives

(36) $${\bar{\pi }}_{jk}=\frac{N_{jk}+\alpha_{0k}-1}{\sum_{i=1}^{M}\frac{\text{det}\left(J_{ij}^{\xi^{-1}}\right)\;\gamma_{ik}\left(\phi_{ij}^{-1}\right)w_{ik}}{\sum_{c=1}^{K}w_{ic}\pi_{jc}}}\;\text{.}$$

Such a solution is then projected onto the constraining hyperplane, by preserving tissue proportions at each voxel

(37) $$\pi_{jk}=\frac{{\bar{\pi }}_{jk}}{\sum_{c=1}^{K}{\bar{\pi }}_{jc}}\;\text{.}$$

Experimental testing of this strategy indicated that it gave a constant improvement of the objective function at a relatively cheap computational cost. Alternatively, iterative constrained non-linear optimisation techniques (Powell, 1978) could have been exploited to solve the template update problem.

#### Computing the deformation fields

Groupwise image alignment is achieved by optimisation of the variational objective function defined in (24), with respect to the parameters used to compute the deformations. This is equivalent to adopting the following image matching or similarity term

(38) $$\begin{array}{l} D=E_{Z}\left[\text{log}p\left(Z|{\text{Θ}}_{\pi },{\text{Θ}}_{w},{\text{Θ}}_{u},{\text{Θ}}_{a}\right)\right]\\ =\sum_{i=1}^{M}\int_{y\in {\text{Ω}}_{i}}\sum_{k=1}^{K}\gamma_{ik}\left(y\right)\text{log}\left(\frac{w_{ik}\pi_{k}\left(\xi_{i}\left(y\right)\right)}{\sum_{c=1}^{K}w_{ic}\;\pi_{c}\left(\xi_{i}\left(y\right)\right)}\right)\text{d}y\;\text{.} \end{array}$$

Additionally, working on discretised image grids, with associated voxel centres ${\left\{y_{ij}\right\}}_{j=1,\ldots ,N_{i}}$, requires reformulating D as

(39) $$D=\sum_{i=1}^{M}\sum_{j=1}^{N_{i}}\sum_{k=1}^{K}\gamma_{ijk}\text{log}\frac{w_{ik}\pi {\text{'}}_{jk}}{\sum_{c=1}^{K}w_{ic}\pi {\text{'}}_{jc}}\;,$$

with

(40) $${\pi^{\prime }}_{jk}=\pi_{k}\left(\xi_{i}\left(y\right)\right){|}_{y=y_{ij}}\;\text{.}$$

The penalty term for this groupwise image registration problem is given by

(41) $$\begin{array}{l} ℛ={ℛ}_{dif}+{ℛ}_{af}=\text{log}p\left({\text{Θ}}_{u}\right)+\text{log}p\left({\text{Θ}}_{a}\right)\\ =-\frac{1}{2}\sum_{i=1}^{M}\left(||L_{u}u_{i}|{|}_{L^{2}}^{2}+a_{i}^{T}\Sigma_{a}^{-1}a_{i}\right)+\text{const}\;, \end{array}$$

with $u_{i}$ being a $3\times N_{i}$ dimensional vector of parameters used for representing the initial velocity field of image *i* and $a_{i}$ encoding affine deformation parameters used to compute the transformation in (19).

For each image *i* in the data set, updating the corresponding initial velocity field, given the current estimates of the templates and all the other model parameters, involves optimising the following objective function

(42) $$\begin{array}{l} E_{dif}^{\left(i\right)}=D^{\left(i\right)}+{ℛ}_{dif}^{\left(i\right)}\\ =\sum_{j=1}^{N_{i}}\sum_{k=1}^{K}\gamma_{ijk}\text{log}\frac{w_{ik}\pi_{k}\left(\xi_{ij}\right)}{\sum_{c=1}^{K}w_{ic}\pi_{c}\left(\xi_{ij}\right)}-\frac{1}{2}||L_{u}u_{i}|{|}_{L^{2}}^{2}\;, \end{array}$$

with respect to $u_{i}$, under the following deformation model

(43) $$\xi_{ij}=\xi_{i}\left(y_{ij}\right)=T_{i}\;\phi_{i}\left(y_{ij}\right)+t_{i}\;,$$

where $\phi_{i}$ is a diffeomorphism computed via geodesic shooting (Ashburner and Friston, 2011) from the corresponding initial velocity field $u_{i}$.

Here image registration is solved via Gauss-Newton optimisation, with a backtracking line search to ensure convergence. This requires computing both the first and second derivatives of the objective function (Hernandez and Olmos, 2008). Such derivatives can be found in Appendix B. This leads to a very high dimensional inverse problem, which unfortunately cannot be solved via numerical matrix inversion, since this would be prohibitively expensive from a computational point of view. The approach adopted in this work consists in treating this optimisation as a partial differential equation problem, which can efficiently be solved using multigrid methods (Modersitzki, 2004). In particular, we adopt the same full multigrid implementation as in Ashburner (2007).

## Validation and discussion

In this section we present results obtained by applying the presented modelling framework to real MR scans acquired with different imaging systems and protocols, as well as to synthetic MR volumes. Both qualitative and quantitative measures will be provided to assess the behaviour of the proposed approach.

All validation analyses have been performed on the full model presented in this paper, since the validity of the individual components (i.e. bias correction, diffeomorphic registration, variational Gaussian mixture modelling) was already assessed in our previous work (Ashburner and Friston, 2005, Ashburner and Friston, 2011, Blaiotta et al., 2016), primarily at a single subject level. Additionally, numerous studies have demonstrated that solving image segmentation and registration tasks in a coupled and iterative manner, as opposed to sequential approaches, ensures more accurate results while reducing the chances of getting prematurely trapped in a local optimum (Ashburner and Friston, 2005, Pohl et al., 2006, Yezzi et al., 2001, Bhatia et al., 2007). This results from the conditional dependencies between the parameters controlling the deformation fields and the intensity distribution parameters, which are used to infer tissue labels. In other words, closely matching the template to each scan is a necessary condition in order to compute accurate segmentations while, in turn, accurate segmentations of the data help to improve alignment with the templates.

### Template construction

We begin validating our approach by illustrating a set of templates obtained by fitting the proposed model to a large neuroimaging data set. Indeed, in remainder of this paper we will be using neuroimaging data for both training and testing, even if, given the generality of the presented approach, applications to many more types of data could be explored.

#### Data

The input data for training the model was obtained from three different databases, two of which are freely accessible for download, thus ensuring that the results presented here could readily be compared to those produced by competing algorithms for medical image registration or segmentation.

*OASIS data set.* The first data set consists of thirty five T1-weighted MR scans from the OASIS (Open Access Series of Imaging Studies) database (Marcus et al., 2007). The data is freely available from the web site http://www.oasis-brains.org, where details on the population demographics and acquisition protocols are also reported. Additionally, the selected thirty five subjects are the same ones that were used within the 2012 MICCAI Multi-Atlas Labeling Challenge (Landman and Warfield, 2012).

*Balgrist data set.* The second data set consists of brain and cervical cord scans of twenty healthy adults, acquired at University Hospital Balgrist with a 3T scanner (Siemens Magnetom Verio). Magnetisation-prepared rapid acquisition gradient echo (MPRAGE) sequences, at 1 mm isotropic resolution, were used to obtain T1-weighted data, while PD-weighted images of the same subjects were acquired with a multi-echo 3D fast low-angle shot (FLASH) sequence, within a whole-brain multi-parameter mapping protocol (Weiskopf et al., 2013, Helms et al., 2008).

*IXI data set.* The third and last data set comprises twenty five T1-, T2-and PD-weighted scans of healthy adults from the freely available IXI brain database, which were acquired at Guy's Hospital, in London, on a 1.5T system (Philips Medical Systems Gyroscan Intera). Additional information regarding the demographics of the population, as well as the acquisition protocols, can be found at http://brain-development.org/ixi-dataset.

The complete data set therefore consists of eighty multispectral scans of healthy adults, obtained with fairly diverse acquisition protocols and using scanning systems produced by different vendors.

Unfortunately, not all the three modalities of interest (T1-, T2-and PD-weighted) are available for all of the subjects. To circumvent the difficulties arising from the presence of missing imaging modalities, without neglecting any of the available data (indeed deletion of entries with missing data is still, in spite of its crudity, a common statistical practice), the Gaussian mixture modelling approach discussed in Blaiotta et al. (2016) was generalised by introducing an additional variational posterior distribution over the missing data points.

In practice, the resulting variational EM scheme iterates over first estimating an approximated posterior distribution on the unknown image intensities, secondly updating the sufficient statistics of the complete (observed and missing) data and finally computing variational posteriors on the Gaussian mixture parameters. Additional computational details relative to this strategy are provided in Appendix C.

In synthesis, it was possible to fit the generative groupwise model described in this paper to the entire data set, in spite of having different imaging modalities available from the different acquisition sites. This is indeed a very common scenario in real life medical imaging problems, therefore it should be actively addressed by processing or modelling solutions that claim to be applicable to large population data (van Tulder and de Bruijne, 2015).

Manual brain labels are freely available for the selected subset of the OASIS data set. Such labels have been generated and made public by Neuromorphometrics, Inc. (http://Neuromorphometrics.com) under academic subscription and they provide a fine parcellation of cortical and non cortical structures, for a total of 139 labels across the brain.

**Fig. 2:**
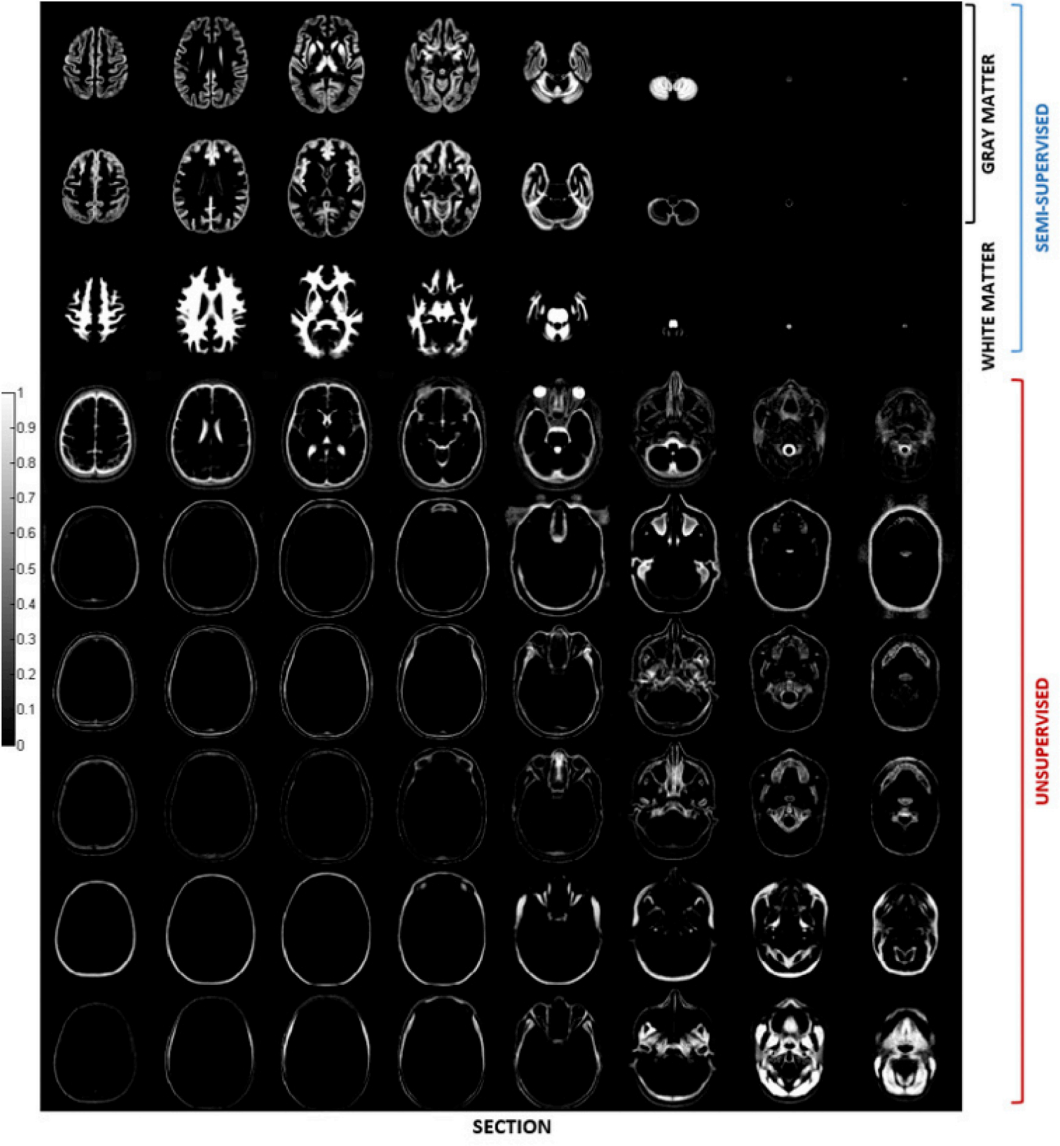
Tissue probability maps obtained by applying the presented groupwise generative model to a multispectral data set comprising head and neck scans of eighty healthy adults, from three different databases.

Part of this label data was used for training of the model while the remainder was left out for testing and validation. In particular, brain labels of twenty out of the thirty five OASIS subjects were used to create gray and white matter ground truth segmentations, which were provided as training input for semisupervised model fitting. In spite of having defined only one gray matter training label, two distinct gray matter classes were introduced in the mixture model (top two rows in Fig. 2), to best capture the corresponding distribution of image intensities, which is poorly represented by a single Gaussian component, as opposed to the distribution of white matter intensities. In this case, posterior membership probabilities were computed making use of equation (17).

Spinal cord manual labels were created for forty subjects (twenty from Balgrist data set and twenty from the IXI data set), by manually delineating the contour of the cord in each transverse slice of the data beginning from the lower extremity of the medulla oblongata. Such labels were randomly split in half for training and half for subsequent test analyses. Due to the limited resolution of the data it was not possible to manually delineate gray and white matter within the spinal cord. For this reason, each voxel classified as spinal cord in the training data was allowed to be assigned either to the gray or to the white matter tissue classes, based on the fit of its intensity value to the underlying Gaussian mixture model, as outlined in equation (17).

#### Tissue templates and intensity priors

The tissue probability maps obtained by applying the modelling framework presented in this paper to the data set described above are depicted in Fig. 2. The total number of tissue classes used for this experiment is equal to twelve but three classes, representing air in the background, are not shown.

The total number of classes was selected based on empirical evidence to obtain a reasonable trade-off between goodness of fit and computational cost. In principle the proposed algorithm would be able to automatically determine the optimal number of classes, as demonstrated in Blaiotta et al. (2016), however this would require setting an initial number of components higher than the unknown optimal one, which, for the size of the data set considered here, would have been computationally very expensive.

Apart from the number of Gaussians, the only hyperparameters that are not estimated from the data but need to be fixed *a priori* are those controlling the bias and registration regularisation. Indeed, one of the strengths of the proposed approach is that, in spite of its complexity most parameters are automatically inferred from the data, thus requiring minimal parameter tuning. For the experiments described in this section we used the settings described below.

The prior distribution on the bias field parameters was assumed to be Gaussian with zero mean and a precision matrix corresponding to a penalty on the Laplacian of the resulting non-uniformity field. Similarly, the affine model was regularised using a Gaussian prior with zero mean and a diagonal covariance matrix, where the magnitude of parameters modelling translations and rotations was less heavily regularised (the prior variance was set equal to 1), compared to that of parameters controlling scaling and shearing, whose prior variance was instead set equal to 0.001. Finally the regularisation settings for diffeomorphic registration were borrowed from the default settings of the Geodesic Shooting toolbox (Ashburner and Friston, 2011) provided with SPM12, which correspond to penalising a linear combination of absolute displacement, membrane energy, bending energy and linear elasticity (more details can be found in the documentation of the SPM12 software).

Fig. 2 shows how one of the two gray matter classes (first row) best fits the subcortical nuclei and also includes voxels affected by partial volume effects at the interface between gray and white matter, while the second one (second row) is more representative of cortical structures, with partial volume effects generated by the mixed presence of gray matter and CSF. The third row in Fig. 2 shows the white matter class, which also includes most of the brainstem and the spinal cord.

The remaining tissue classes were estimated in a purely unsupervised way. Therefore a non ambiguous anatomical interpretation is not straightforward.

Tissue class four (fourth row) mainly contains CSF, even if other tissues are also present, especially in the neck area. This should be attributed to the lack of CSF training labels as well as to a poor multivariate coverage of the cervical region in the available data. In fact, data from the OASIS set is truncated around the first cervical vertebra. The T1-weighted scans of the IXI data set cover up to the C2/C3 vertebral level, but the corresponding T2-and PD-weighted scans do not extend beyond the brainstem. Indeed, only the data from the second database (Balgrist hospital) provides more than one modality covering up to around the fourth cervical vertebra. In this case though, additional difficulties arose from poor inter-modality alignment of the data, a problem that turned out to be particularly severe in the cervical region and that, given its non-linearity, cannot be fully compensated for by affine inter-modality coregistration. This result confirms the importance and usefulness of non-linear image coregistration tools, particularly when modelling highly deformable anatomical structures (Stroman et al., 2008, Fonov et al., 2014). Such a problem was not addressed in this paper, which limits the applicability of the proposed approach when scans of the same subject are not in good alignment. However it would in principle be possible to introduce inter-modality deformation fields within the same generative model adopted here and we aim to address this issue as part of our future work.

Bone tissue is also not easily identifiable from the data available for this experiment, but it could have potentially been much better extracted by incorporating some CT scans into the training data.

Fat and soft tissues are mainly represented in the last two classes (bottom two rows in Fig. 2).

While quantitative evaluation analyses of the proposed modelling approach will be reported in the following sections, the results presented in Fig. 2 provide a qualitative insight into the performance of the algorithm discussed in this paper. In particular, the sharp appearance of the tissue probability maps suggests that the proposed model can capture a significant amount of anatomical variability thus ensuring fine alignment of complex anatomical structures, whose shape varies significantly across individuals. In turn, this represents a very valuable property for the purpose of performing statistical group analyses on neuroimaging data, since most of these studies rely on the assumption that accurate anatomical and functional correspondences can be estimated through some form shape mapping procedure, which is most commonly implemented using non-linear image registration techniques.

**Fig. 3:**
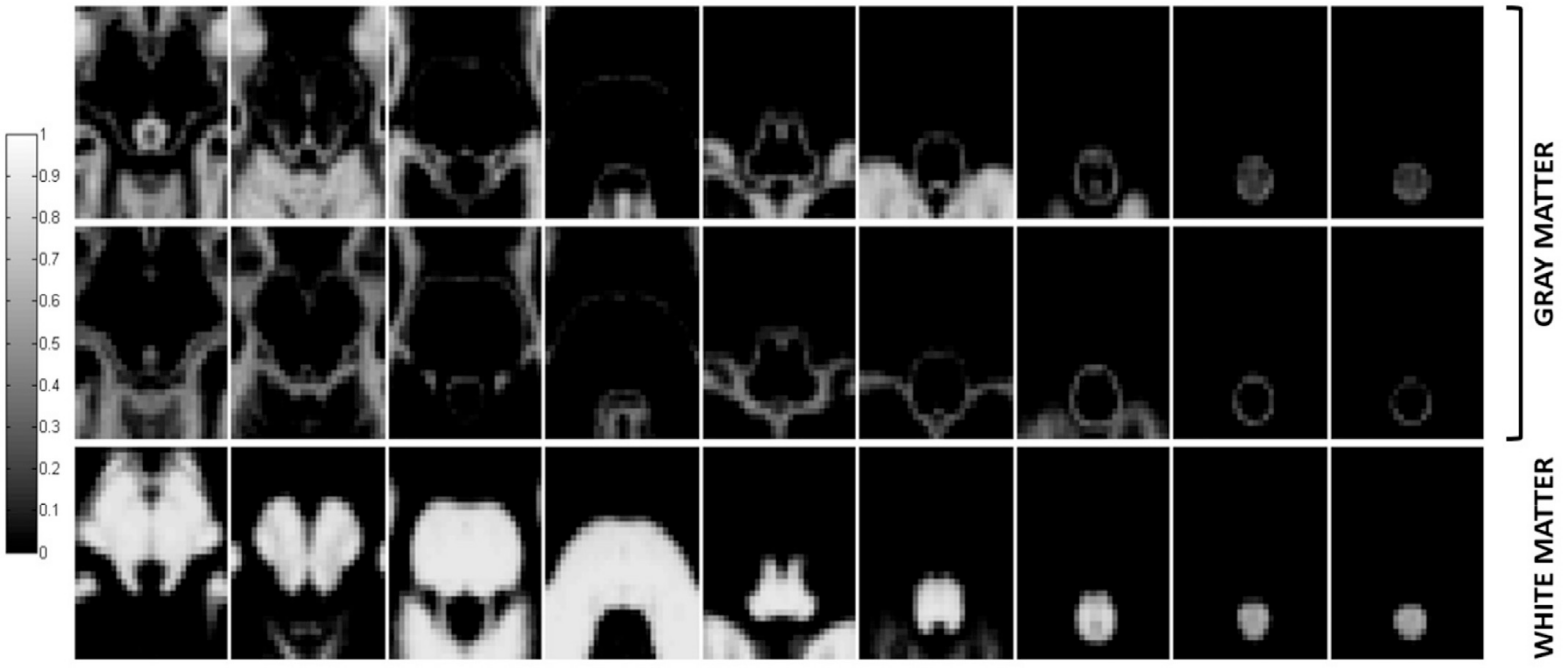
Zoomed views of the gray (top two rows) and white matter (bottom row) tissue probability maps at the brainstem and spinal cord levels. Each transverse slice covers an area of $45\times 53{\text{ mm}}^{2}$, while the axial distance between adjacent slices along each row equals 7.5 mm. The order of tissue classes from top to bottom is the same as in the top three rows of Fig. 2, however more densely spaced transverse slices are depicted here.

Fig. 3 illustrates zoomed views of the gray and white matter tissue probability maps at the brainstem and spinal cord levels.

**Fig. 4:**
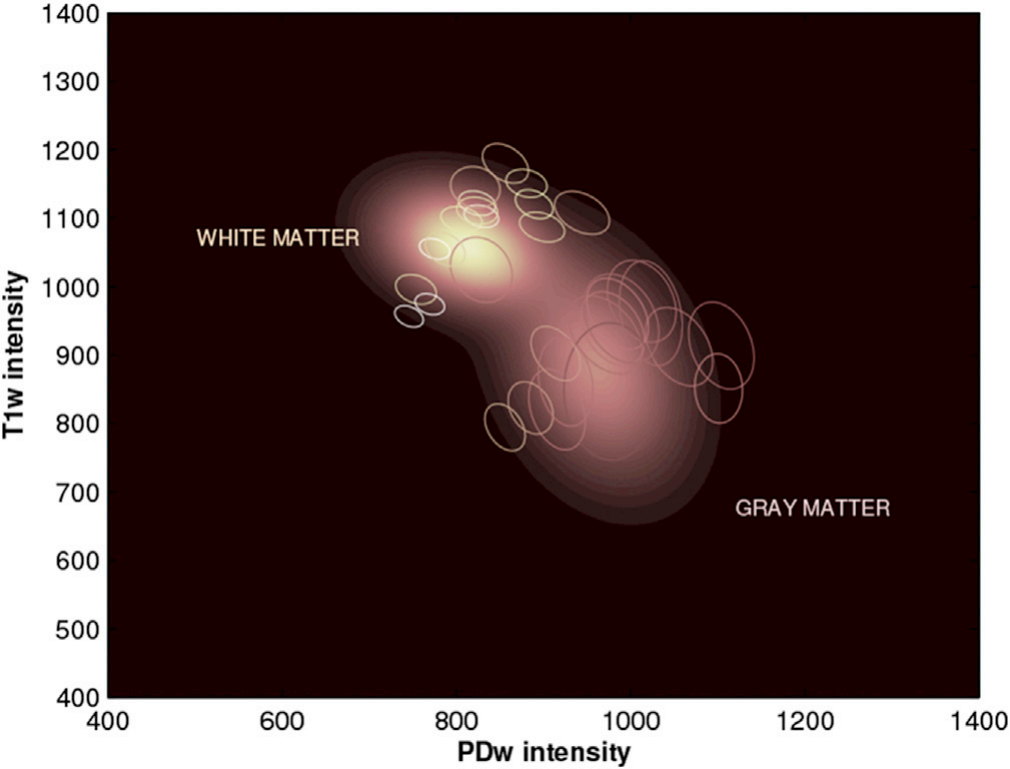
Prior distribution over the mean intensity of gray and white matter, in T1-and PD-weighted data. Contour plots illustrate a number of randomly selected individual posteriors.

The empirical Bayes learning procedure, introduced in Blaiotta et al. (2016) to estimate suitable prior distributions for the parameters of the Gaussian mixture model, was applied here to the same data used to construct the templates. Some of the results are summarised in Fig. 4, where the estimated empirical prior distributions on the mean intensity of gray and white matter are depicted, with overlaid contour plots showing some of the individual posteriors (randomly selected across the entire population).

Such results indicate that the proposed empirical Bayes learning scheme can serve to capture, not only the variability of mean tissue intensity across subjects for each of the modalities of interest, but also the amount of covariance between such modalities. Information of this sort can potentially be used in a number of different frameworks, for solving problems such as tissue segmentation, pathology detection or image synthesis.

**Fig. 5:**
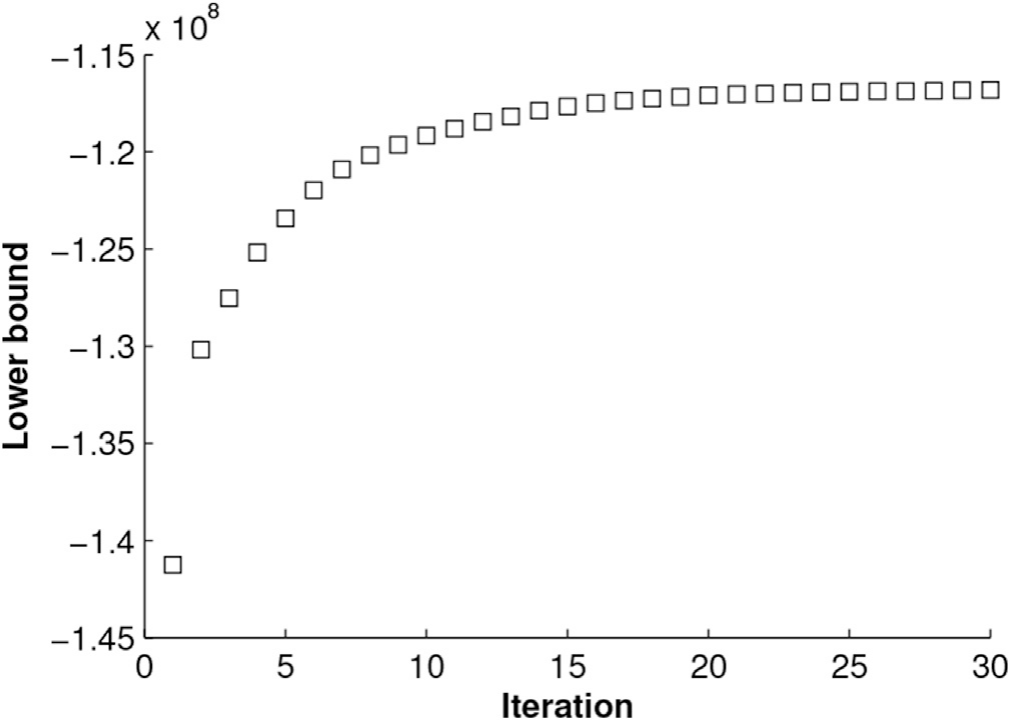
Lower bound values as a function of iteration number during model training.

In Fig. 5 we report a plot of the lower bound during model fitting as a qualitative demonstration of convergence.

Run time for the data set presented here was around 50 h on an 8 Core PC at 3 GHz with 32 GB RAM.

#### Validity of groupwise registration

The performance of groupwise registration achieved by the presented algorithm was assessed by computing pairwise overlap measures for all possible couples of spatially normalised test label maps (i.e. label maps that were not used for training the model), which were obtained by applying the inverse of the estimated deformation fields to the maps in their native space. The Dice^4^ score coefficient was chosen as a metric of similarity.

**Fig. 6:**
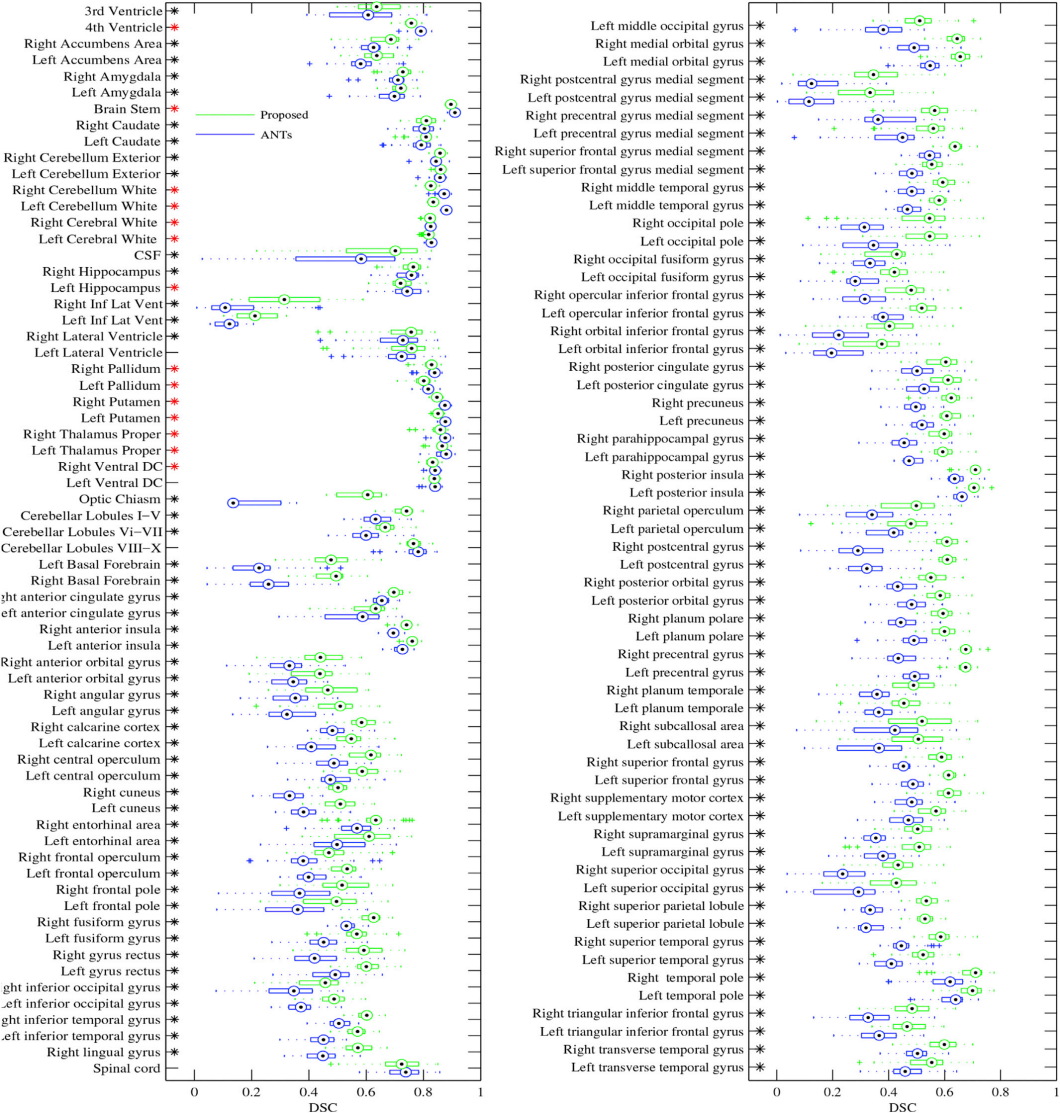
Registration accuracy achieved by the presented method, compared to the performance of the diffeomorphic groupwise template construction method distributed with ANTs. Boxplots, in green for the proposed method and blue for ANTs, illustrate the distributions of overlap measures (Dice score coefficients) obtained by considering all possible pairs of spatially normalised test images, whose labels were not used for model fitting. Stars indicate statistically significant differences between the two methods, assessed by means of paired t-tests without correcting for multiple comparisons. Star colours encode the method that achieved higher performance (black for the proposed algorithm and red for ANTs).

Results are summarised in Fig. 6, where the accuracy of the algorithm presented here is compared to that achieved by the groupwise image registration method described in Avants et al. (2010), whose implementation is publicly available, as part of the Advanced normalisation Tools (ANTs) package, through the web site http://stnava.github.io/ANTs/. Indeed, the symmetric diffeomorphic registration framework implemented in ANTs has established itself as the state-of-the-art of medical image nonlinear spatial normalisation (Klein et al., 2009).

A number of options can be customised within the template construction framework distributed with ANTs. The experiments, whose results are reported here, were performed with the settings recommended in the package documentation for brain MR data, which are also reported in Table 2. The same strategy outlined above was applied in order to compute Dice score coefficients for all structural labels and all possible pairs of test images.

**Table 2:** Options selected to perform groupwise registration with ANTs, using the antsMultivariateTemplateConstruction script provided with the ANTS package.

Option	Value
Similarity Metric	Cross-correlation (CC)
Transformation model	Greedy SyN (GR)
Initial rigid body	yes
N4 Bias Correction	yes
Number of resolution levels	4
Number of iterations	100×70×50×10
Gradient step	0.2
Number of template updates	4

Results of this validation analyses indicate that the method presented here, in spite of not being as accurate as ANTs for aligning some subcortical brain structures (e.g. thalamus, putamen, pallidum and brainstem), provided significantly better overlap when registering cortical regions, as assessed by means of paired t-tests with a significance threshold of 0.05 and without correcting for multiple comparisons.

#### Accuracy of tissue classification

The accuracy of tissue classification achieved by the method presented in this paper was first evaluated on test data that was used to create the templates but without providing manual labels for training the model. The aim in this case is to determine to which extent the proposed method can capture significant features of the training data, by learning from few annotated examples.

Dice scores were computed to compare the automated segmentations produced via semisupervised groupwise model fitting, with the ground truth, obtained by merging all the gray and white matter brain structures (labels) into two tissue classes respectively. The probabilistic gray and white matter segmentations produced by the proposed algorithm were thresholded at 0.5, in order to obtain binary label maps, directly comparable to the ground truth. Results are summarised in seg, which shows the distributions of Dice scores obtained for gray and white matter.

Such results were then compared to those produced by the brain segmentation algorithm implemented in SPM12, using the tissue probability maps illustrated in Fig. 2. Indeed the proposed approach extends and generalises the model underlying SPM12 segmentation method. This is achieved by replacing a small deformation approach to image registration with a diffeomorphic representation of shapes, as well as by introducing empirical intensity priors (within a variational Bayes framework) and by allowing learning of the tissue probability maps directly from the data.

**Fig. 7:**
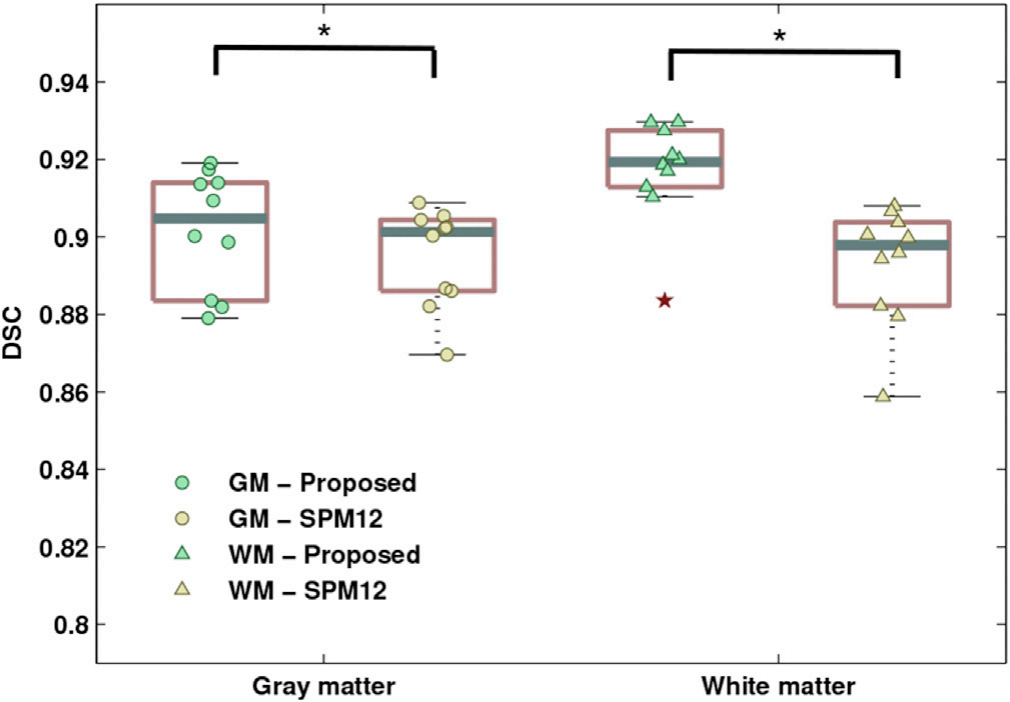
Brain segmentation accuracy of the presented method in comparison to SPM12 image segmentation algorithm. Boxplots indicate the distributions of Dice score coefficients, with overlaid scatter plots of the estimated scores. Red stars denote outliers.

Therefore, results of these analyses, which are also shown in Fig. 7, provide a direct insight into the performance gain achieved by incrementing the model available in SPM12 as indicated above. In particular, our experiments indicate that the proposed method enables higher tissue classification accuracy, for both gray and white matter (assessed by means of paired t-tests with a significance threshold of 0.05).

The experiments described in this section however did not test the generalisation capability of the proposed method, which would have required a k-fold cross-validation design in order to exploit as much as possible of the available data during training. Unfortunately this was highly unpractical in this case due to the expensive computational cost of groupwise model fitting. The generalisation performance of the proposed model is instead assessed in the next section, using a hold-out approach and making use of both real and synthetic unseen brain MR data, for which tissue labels are known.

### Modelling unseen data

Further validation experiments were performed to quantify the accuracy of the framework described in this paper to model unseen data, that is to say data that was not included in the atlas generation process.

In particular, we evaluated registration accuracy using data from the Internet Brain Segmentation Repository (IBSR), which is provided by the Centre for Morphometric Analysis at Massachusetts General Hospital (http://www.cma.mgh.harvard.edu/ibsr/). Experiments to assess bias correction and segmentation accuracy were instead performed on synthetic T1-weighted brain MR scans from the Brainweb database (http://brainweb.bic.mni.mcgill.ca/), which were simulated using a healthy anatomical model under different noise and bias conditions.

#### Accuracy of registration

To assess the performance of the presented method for spatially normalising unseen test data we made use of the ISBR data set, which consists of 18 T1-weighted brain images with manual labels of 84 anatomical structures. Such a data set was also used by Klein et al. (2009) for their evaluation of 14 brain image registration algorithms. Therefore the results presented in the remainder of this section allow comparing the proposed approach to a number of image registration methods available to the neuroimaging community.

For this experiment we registered each of the 18 unseen scans to the tissue probability maps presented in Fig. 2 by adopting the algorithmic framework presented in this paper. We then computed target overlap measures for all pairs of spatially normalised images. This was achieved by composing the inverse of each estimated deformation field with every other direct transformation and applying the resulting warps to the labels in their native space, so as to map between each couple of subjects. Our method, after averaging across different brain regions, achieved a median overlap score of 0.54 with the 25th and 75th percentiles equal to 0.51 and 0.57 respectively. Instead, the best performing method in the experiments performed by Klein et al. (2009) obtained a median score of 0.55 on the same data set.

Such results indicate that our approach, in spite of being intrinsically best suited for groupwise analyses, where the reference image is constructed iteratively from the same scans that are being spatially normalised (Avants et al., 2010), can provide accurate results also when mapping test data to a fixed reference that encodes the average shape of a different population. Indeed, this experiment was intended as a means to assess the generalisation capability of the templates constructed with the proposed approach, while ensuring a fairer comparison to the results of Klein et al. (2009), which were produced via pairwise non-linear registration rather than by estimating group averages.

#### Accuracy of bias correction

A healthy adult brain MR model was processed by means of the algorithm discussed here, using the brain and spinal cord templates previously constructed as tissue priors. Different levels of noise and bias field were added to the uncorrupted synthetic data, to test the behaviour of the proposed modelling scheme in different noise (1%, 3%, 7%) and bias conditions (20% and 40%).

The noise in these simulated images has Rayleigh statistics in the background and Rician statistics in the signal regions and its level is computed as a percent standard deviation ratio, relative to the MR signal, for a reference tissue (Cocosco et al., 1997).

Regarding the bias field instead, 20% bias is modelled as a smooth field in the range [0.9, 1.1] while 40% bias is obtained by rescaling of the 20% field, so as to range between 0.8 and 1.2.

Table 3 reports the Pearson product-moment correlation coefficients between the ground truth and the estimated bias fields, for the different bias ranges and noise levels. Results indicate that the similarity between the estimated and true bias decreases for more intense non-uniformity fields and higher noise levels.

**Table 3:** Pearson's correlation coefficients between the ground truth bias fields and those estimated by the presented algorithm, for simulated T1-weighted data.

**Bias**	**Noise**		
	**1%**	**3%**	**7%**
**20%**	0.86	0.86	0.70
**40%**	0.72	0.72	0.51

Indeed this is not surprising, as the penalty term, which enforces smoothness of the bias field, has a greater impact in determining the shape of the estimated bias when the non-uniformity fields have a larger dynamic range. Nevertheless, results reported in the following section will show how this increased mismatch between the estimated and true bias, for higher non-uniformities, does not seem to affect the accuracy of tissue segmentation. On the other hand, the accuracy of bias correction is directly related to the amount of noise corrupting the data, mainly due to how this affects the precision associated with estimation of the Gaussian mixture parameters. For a comparison of these results with the performance of SPM12 bias correction on simulated T1-weighted scans from the Brainweb database see Blaiotta et al. (2016).

#### Accuracy of tissue classification

For the same synthetic data the accuracy of tissue classification was also evaluated, by comparing the similarity between the estimated gray and white matter segmentations and the underlying anatomical model.

**Fig. 8:**
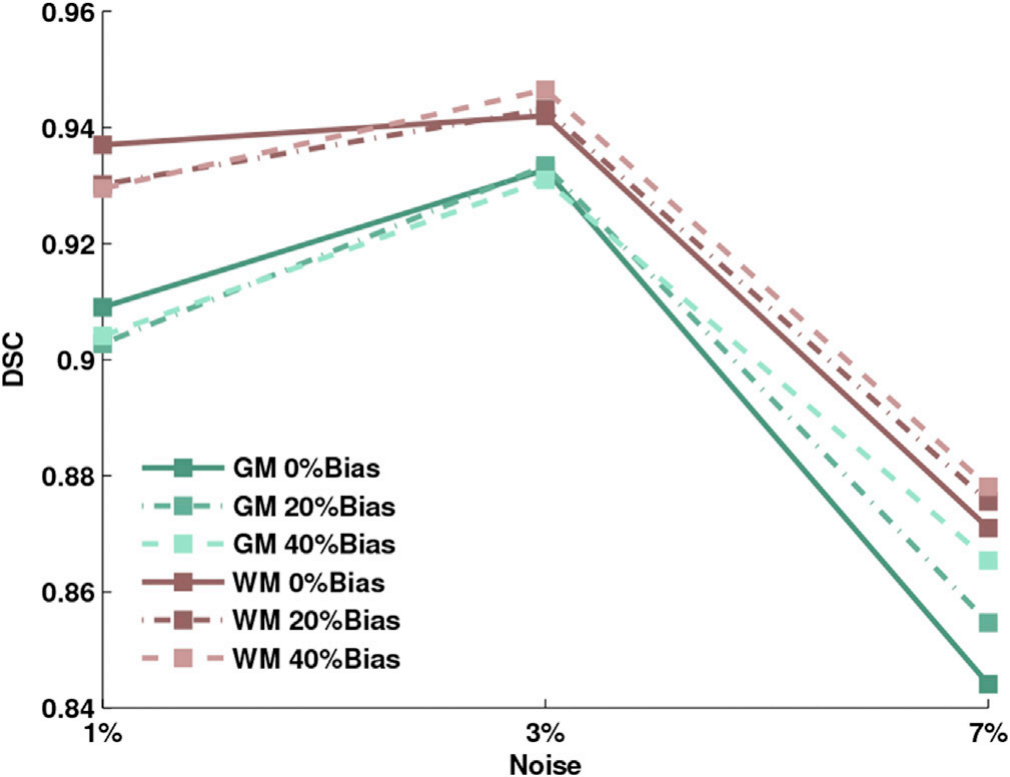
Dice scores between the estimated and ground truth segmentations for brain white matter and brain gray matter, under different noise and bias conditions, for synthetic T1-weighted data.

Results are reported in Fig. 8, which shows the Dice score coefficients obtained under different bias and noise conditions.

The Brainweb database has been extensively used in the neuroimaging community to validate MR image processing algorithms. Therefore the results reported here should be directly comparable to the performance of many brain segmentation techniques present in the literature.

## Conclusions

This paper presented a comprehensive generative modelling framework, suitable to capture anatomical variability from large cross-sectional MR data sets. From a theoretical perspective, such a framework relies on variational probability density estimation techniques to model the observed data (i.e. MR image intensities). Additionally, a hierarchical modelling perspective is proposed, where observations from a population of subjects are used to construct empirical intensity priors, which can then serve to inform models of new data. Shape modelling is performed via groupwise diffeomorphic registration, thus ensuring bijective (i.e. one-to-one) differentiable mappings between anatomical configurations (Miller, 2004). Such an approach enables a rigorous mathematical encoding of anatomical shapes via deformable template matching (Christensen et al., 1996), therefore providing a quantitative framework for the analysis of shape variation and covariation.

Data for validating the method was collected from many different brain databases, most of which are publicly accessible to the research community. Results of our experiments, performed both on training and unseen test data, indicate that the proposed approach can accurately align complex anatomical shapes, such as the brain and spinal cord, and segment data into tissue types, while being robust to inter-scanner signal variations. Therefore, the proposed algorithm defines a convenient framework to extract volumetric and morphometric information from large structural neuroimaging data sets, in a fully automated manner. At the same time it provides outputs that can be readily interpreted, for instance via statistical hypothesis testing, with the ultimate goal of comparing different populations, treatment effects etc. (Ashburner and Friston, 2000).

Additionally, our results suggest that the proposed approach could be useful to construct templates capable of capturing the peculiar anatomical features of populations poorly represented by standard anatomical atlases (such as young or elderly populations, diseased populations, or individuals belonging to different ethnic groups (Tang et al., 2010, Fillmore et al., 2015)). This would not only lead to more accurate segmentation results, but as a direct consequence, also increase the reliability of subsequent data analyses, which build models of the segmented data to infer or predict clinically meaningful information.

It should be noted that, in spite of having been tested on neuroimaging data sets, our method was intentionally formulated as a general approach, which makes it potentially suitable to solve a wide range of imaging problems, for instance in the context of animal imaging studies or for the analysis of different human organs using multispectral MR data sets. We aim to explore some of this applications as part of our future work.

## Appendix A Derivatives of the lower bound with respect to the affine parameters

The affine parameters, for each subject *i*, can be estimated (i.e. optimised) in a Gauss-Newton fashion, so as to maximise of the following objective function

(A.1) $$\begin{array}{l} E_{af}^{\left(i\right)}=D^{\left(i\right)}+{ℛ}_{af}^{\left(i\right)}\\ =\overset{N_{i}}{\sum_{j=1}}\overset{K}{\sum_{k=1}}\gamma_{ijk}\text{log}\frac{w_{ik}\pi_{k}\left(\xi_{ij}\right)}{\sum_{c=1}^{K}w_{ic}\pi_{c}\left(\xi_{ij}\right)}-\frac{1}{2}a_{i}^{T}\Sigma_{a}^{-1}a_{i}\;, \end{array}$$

with respect to $a_{i}$.

The gradients and Hessians, which are useful to solve this problem are reported below. In particular, for the matching term, the following derivatives need to be computed

(A.2) $$\frac{\partial D^{\left(i\right)}}{\partial a_{i}}=\overset{N_{i}}{\sum_{j=1}}\overset{K}{\sum_{k=1}}\left(\gamma_{ijk}-\frac{w_{ik}\;\pi_{k}\left(\xi_{ij}\right)}{\sum_{c=1}^{K}w_{ic}\;\pi_{c}\left(\xi_{ij}\right)}\right)\;g_{jk}^{\pi }\;,$$

where $g_{jk}^{\pi }$ is defined as

(A.3) $$g_{jk}^{\pi }=B_{i}^{T}\left(\left[\phi_{ij}\;,1\right]⊗\nabla \left[\text{log}\left(\pi_{k}\left(\xi_{ij}\right)\right)\right]\right)\;,$$

with

(A.4) $$B_{i}^{T}=\frac{\partial S_{i}}{\partial a_{i}}\;,$$

and

(A.5) $$S_{i}=\left[\begin{array}{cc} T_{i} & t_{i}\\ 0 & 1 \end{array}\right]\;\text{.}$$

(A.6) $$\begin{array}{l} \frac{\partial^{2}D^{\left(i\right)}}{\partial a_{i}^{2}}=\overset{N_{i}}{\sum_{j=1}}\left(\overset{K}{\sum_{k=1}}\frac{w_{ik}\;\pi_{k}\left(\xi_{ij}\right)}{\sum_{c=1}^{K}w_{ic}\;\pi_{c}\left(\xi_{ij}\right)}\;g_{jk}^{\pi }\right)\\ \times {\left(\overset{K}{\sum_{k=1}}\frac{w_{ik}\left(\pi_{k}\left(\xi_{ij}\right)\right)}{\sum_{c=1}^{K}w_{ic}\left(\pi_{k}\left(\xi_{ij}\right)\right)}\;g_{jk}^{\pi }\right)}^{T}\\ -\overset{N_{i}}{\sum_{j=1}}\overset{K}{\sum_{k=1}}\frac{w_{ik}\;\pi_{k}\left(\xi_{ij}\right)}{\sum_{c=1}^{K}w_{ic}\;\pi_{c}\left(\xi_{ij}\right)}\;g_{jk}^{\pi }{\left(g_{jk}^{\pi }\right)}^{T}\;\text{.} \end{array}$$

Gradients and Hessians of the penalty term are instead given by

(A.7) $$\frac{\partial {ℛ}_{af}^{\left(i\right)}}{\partial a_{i}}=-\Sigma_{a}^{-1}a_{i}\;,$$

(A.8) $$\frac{\partial^{2}{ℛ}_{af}^{\left(i\right)}}{\partial a_{i}^{2}}=-\Sigma_{a}^{-1}\;\text{.}$$

## Appendix B Derivatives of the lower bound with respect to the initial velocities

Optimisation of the initial velocities, for each image *i*, requires maximising the following objective function

(B.1) $$\begin{array}{l} E_{dif}^{\left(i\right)}=D^{\left(i\right)}+{ℛ}_{dif}^{\left(i\right)}\\ =\overset{N_{i}}{\sum_{j=1}}\overset{K}{\sum_{k=1}}\gamma_{ijk}\text{log}\frac{w_{ik}\pi_{k}\left(\xi_{ij}\right)}{\sum_{c=1}^{K}w_{ic}\pi_{c}\left(\xi_{ij}\right)}-\frac{1}{2}\overset{M}{\sum_{i=1}}||L_{u}u_{i}|{|}_{L^{2}}^{2}\;, \end{array}$$

with respect to $u_{i}$.

Here, we report the first and second derivatives of this objective function, which are useful to solve the registration problem using gradient-based techniques, such as the Gauss-Newton algorithm.

The gradient of the matching term $D^{\left(i\right)}$ with respect to $u_{i}$ is given by

(B.2) $$\begin{array}{l} \frac{\partial D^{\left(i\right)}}{\partial u_{i}}=\overset{K}{\sum_{k=1}}\gamma_{ijk}\frac{\partial }{\partial u_{i}}\left(\text{log}\frac{w_{ik}\;\pi_{k}\left(\xi_{i}\right)}{\sum_{c=1}^{K}w_{ic}\;\pi_{c}\left(\xi_{i}\right)}\right)\\ =\overset{K}{\sum_{k=1}}\gamma_{ijk}\;\left(g_{k}^{\pi }-\overset{K}{\sum_{c=1}}\frac{w_{ic}\;\pi_{c}\left(\xi_{i}\right)}{\sum_{c=1}^{K}w_{ic}\;\pi_{c}\left(\xi_{i}\right)}g_{c}^{\pi }\right)\;, \end{array}$$

which, making use of $\sum_{k=1}^{K}\gamma_{ijk}=1\;$, can be rewritten as

(B.3) $$\frac{\partial D^{\left(i\right)}}{\partial u_{i}}=\overset{K}{\sum_{k=1}}\left(\gamma_{ik}-\frac{w_{ik}\;\pi_{k}\left(\xi_{i}\right)}{\sum_{c=1}^{K}w_{ic}\;\pi_{c}\left(\xi_{i}\right)}\right)\;g_{k}^{\pi }\;,$$

where $g_{k}^{\pi }$ is computed, at each voxel *j*, by

(B.4) $$g_{jk}^{\pi }={\left(T_{i},J_{ij}^{\xi }\right)}^{T}\nabla \left[\text{log}\left(\pi_{k}\left(\xi_{ij}\right)\right)\right]\;,$$

and $J_{i}^{\xi }$ indicates the Jacobian matrix of $\xi_{ij}$.

An approximated positive semidefinite Hessian of D can instead be computed by discarding the second derivatives of the logarithm of tissue priors

(B.5) $$\frac{\partial^{2}}{\partial y^{2}}\text{log}\left(\frac{w_{ik}\left(\pi_{k}\left(\xi_{i}\left(y\right)\right)\right)}{\sum_{c=1}^{K}w_{ic}\left(\pi_{c}\left(\xi_{i}\left(y\right)\right)\right)}\right)=0\;,\forall y\in {\text{Ω}}_{i}\;,$$

to give

(B.6) $$\begin{array}{l} \frac{\partial^{2}D^{\left(i\right)}}{\partial u_{i}^{2}}=\left(\overset{K}{\sum_{k=1}}\frac{w_{ik}\;\pi_{k}\left(\xi_{i}\right)}{\sum_{c=1}^{K}w_{ic}\;\pi_{c}\left(\xi_{i}\right)}\;g_{k}^{\pi }\right)\\ \times {\left(\overset{K}{\sum_{k=1}}\frac{w_{ik}\left(\pi_{k}\left(\xi_{i}\right)\right)}{\sum_{c=1}^{K}w_{ic}\left(\pi_{k}\left(\xi_{i}\right)\right)}\;g_{k}^{\pi }\right)}^{T}\\ -\overset{K}{\sum_{k=1}}\frac{w_{ik}\;\pi_{k}\left(\xi_{i}\right)}{\sum_{c=1}^{K}w_{ic}\;\pi_{c}\left(\xi_{i}\right)}\;g_{k}^{\pi }{\left(g_{k}^{\pi }\right)}^{T}\;\text{.} \end{array}$$

Finally, the first and second derivatives of the penalty term ℛ, which are also required to optimise (B.1), can be computed by

(B.7) $$\frac{\partial {ℛ}_{dif}^{\left(i\right)}}{\partial u_{i}}=-L_{u}^{†}L_{u}u_{i}\;,$$

(B.8) $$\frac{\partial^{2}{ℛ}_{dif}^{\left(i\right)}}{\partial u_{i}^{2}}=-L_{u}^{†}L_{u}\;\text{.}$$

## Appendix C Variational Gaussian mixtures: inference of missing data

The variational Bayes EM algorithm for fitting Gaussian mixture models, described in Blaiotta et al. (2016), can be generalised to handle the case where some components of the *D*-dimensional observation $x_{j}$ are missing.

Having denoted

(C.1) $$x_{j}=\left[\begin{array}{c} o_{j}\\ h_{j} \end{array}\right]\;,$$

with $o_{j}$ being the observed data and $h_{j}$ the missing (hidden) data, the Gaussian likelihood $p\left(x_{j}|z_{jk}=1,\mu_{k},\Sigma_{k}\right)$ can be expressed as

(C.2) $$p\left(x_{j}|z_{jk}=1,\mu_{k},\Lambda_{k}\right)=N\left(\left[\begin{array}{c} o_{j}\\ h_{j} \end{array}\right]|\left[\begin{array}{c} \mu_{k}^{o}\\ \mu_{k}^{h} \end{array}\right]\;,{\left[\begin{array}{cc} \Lambda_{k}^{o,o} & \Lambda_{k}^{o,h}\\ \Lambda_{k}^{o,h} & \Lambda_{k}^{h,h} \end{array}\right]}^{-1}\right)\text{,}$$

by making use of block matrix notation to partition the mean vector $\mu_{k}$ and the precision matrix $\Lambda_{k}$.

In this case $h_{j}$ is treated as an unobserved random variable. Thus, in a variational Bayes setting, an additional posterior factor can be introduced for each missing data point $h_{j}$ to give

(C.3) $$\begin{array}{l} q\left(H,Z,{\text{Θ}}_{\mu },{\text{Θ}}_{\text{Σ}}\right)=q\left(H\right)q\left(Z\right)q\left({\text{Θ}}_{\mu },{\text{Θ}}_{\text{Σ}}\right)\\ =q\left(Z\right)q\left({\text{Θ}}_{\mu },{\text{Θ}}_{\text{Σ}}\right)\overset{N}{\prod_{j=1}}q\left(h_{j}\right)\;\text{.} \end{array}$$

Making use of the general result $q_{\hat{s}}\left({\text{Θ}}_{\hat{s}}\right)\propto \text{exp}\left(E_{s\neq \hat{s}}\left[\text{log}p\left(X,\text{Θ}\right)\right]\right)$ (Bishop, 2006), an approximated posterior on the missing data point $h_{j}$ can be computed by

(C.4) $$\begin{array}{l} \text{log}q\left(h_{j}\right)=E_{Z,{\text{Θ}}_{\mu },{\text{Θ}}_{\text{Σ}}}\left[\text{log}p\left(x_{j},z_{j},{\text{Θ}}_{\mu },{\text{Θ}}_{\text{Σ}}|{\text{Θ}}_{\pi }\right)\right]+\text{const}\\ =E_{Z,{\text{Θ}}_{\mu },{\text{Θ}}_{\text{Σ}}}\left[\text{log}p\left(x_{j}|z_{j},{\text{Θ}}_{\mu },{\text{Θ}}_{\text{Σ}}\right)\right]+E_{Z}\left[\text{log}p\left(z_{j}|{\text{Θ}}_{\pi }\right)\right]\\ +E_{{\text{Θ}}_{\mu },{\text{Θ}}_{\text{Σ}}}\left[\text{log}p\left({\text{Θ}}_{\mu },{\text{Θ}}_{\text{Σ}}\right)\right]+\text{const}\;, \end{array}$$

where ${\text{Θ}}_{\pi }$ denotes the mixing proportion parameter set, treated here via maximum likelihood, and $p\left({\text{Θ}}_{\mu },{\text{Θ}}_{\text{Σ}}\right)$ is a conjugate Gaussian-Wishart prior on the means and covariances of the model.

Ignoring the terms independent from $h_{j}$, equation (C.4) can be rewritten as

(C.5) $$\begin{array}{l} \text{log}q\left(h_{j}\right)=\overset{K}{\sum_{k=1}}\gamma_{jk}\;E_{{\text{Θ}}_{\mu },{\text{Θ}}_{\text{Σ}}}\left[\text{log}N\left(x_{j}|\mu_{k},\Sigma_{k}\right)\right]+\text{const}\\ =\frac{1}{2}\overset{K}{\sum_{k=1}}\gamma_{jk}h_{j}^{T}E_{{\text{Θ}}_{\mu },{\text{Θ}}_{\text{Σ}}}\left[\Lambda_{k}^{h,h}\right]h_{j}\\ +\overset{K}{\sum_{k=1}}\gamma_{jk}h_{j}^{T}E_{{\text{Θ}}_{\mu },{\text{Θ}}_{\text{Σ}}}\left[\Lambda_{k}^{o,h}\right]\left(o_{j}-E_{{\text{Θ}}_{\mu },{\text{Θ}}_{\text{Σ}}}\left[\mu_{k}^{o}\right]\right)\\ -\overset{K}{\sum_{k=1}}\gamma_{jk}h_{j}^{T}E_{{\text{Θ}}_{\mu },{\text{Θ}}_{\text{Σ}}}\left[\Lambda_{k}^{h,h}\right]E_{{\text{Θ}}_{\mu },{\text{Θ}}_{\text{Σ}}}\left[\mu_{k}^{h}\right]+\text{const}\;\text{.} \end{array}$$

The previous equation indicates that the unobserved value $h_{j}$ is drawn from a Gaussian mixture distribution with mixing proportions equal to the posterior (after having observed $o_{j}$) membership probabilities ${\left\{\gamma_{jk}\right\}}_{k=1,\ldots ,K}$, while the Gaussian means ${\left\{n_{jk}\right\}}_{k=1,\ldots ,K}$ and covariances ${\left\{P_{jk}\right\}}_{k=1,\ldots ,K}$ are given by

(C.6) $$\begin{array}{l} n_{jk}=E_{{\text{Θ}}_{\mu },{\text{Θ}}_{\text{Σ}}}\left[\mu_{k}^{h}\right]+{\left(E_{{\text{Θ}}_{\mu },{\text{Θ}}_{\text{Σ}}}\left[\Lambda_{k}^{h,h}\right]\right)}^{-1}\\ \times E_{{\text{Θ}}_{\mu },{\text{Θ}}_{\text{Σ}}}\left[\Lambda_{k}^{o,h}\right]\left(E_{{\text{Θ}}_{\mu },{\text{Θ}}_{\text{Σ}}}\left[\mu_{k}^{o}\right]-o_{j}\right)\;, \end{array}$$

(C.7) $$P_{k}=E_{{\text{Θ}}_{\mu },{\text{Θ}}_{\text{Σ}}}\left[\Lambda_{k}^{h,h}\right]\;\text{.}$$

Given the posteriors q(Z) and q(H), the following sufficient statistics of X can be computed

(C.8) $$s_{1k}=\left[\begin{array}{c} \overset{N}{\sum_{j=1}}\gamma_{jk}o_{j}\\ \overset{N}{\sum_{j=1}}\gamma_{jk}n_{jk} \end{array}\right]\;,$$

(C.9) $$S_{2k}=\left[\begin{array}{cc} \overset{N}{\sum_{j=1}}\gamma_{jk}o_{j}o_{j}^{T} & \overset{N}{\sum_{j=1}}\gamma_{jk}o_{j}n_{jk}^{T}\\ \overset{N}{\sum_{j=1}}\gamma_{jk}n_{jk}o_{j}^{T} & \overset{N}{\sum_{j=1}}\gamma_{jk}\left(n_{k}n_{jk}^{T}+{\left(P_{k}\right)}^{-1}\right) \end{array}\right]\;\text{.}$$

Once such sufficient statistics have been evaluated, they can be used to update the Gaussian-Wishart posteriors $q\left({\text{Θ}}_{\mu },{\text{Θ}}_{\text{Σ}}\right)$ in the exact same way as in Blaiotta et al. (2016). Such posteriors are in turn used to compute the expectations that appear in equation (C.6), (C.7), in an iterative EM fashion.

An inner product naturally induces a norm by $||x||={\langle x,x\rangle }^{1/2}$, therefore every inner product space is also a normed vector space (Dieudonné, 2013).

## Footnotes

1 Our formulation tries to be as general as possible, therefore we do not assume that different images will have the same size, as this often does not occur in practice. The size of the template is calculated so as to cover the field of view of all the images in the data set.

2 A Riemannian manifold, in differential geometry, is a smooth manifold *M* equipped with a Riemannian metric (inner product). In particular, the Riemannian metric $G_{p}$ on the *n*-dimensional manifold $M^{n}$ defines, for every point p∈M, the scalar product of vectors in the tangent space $T_{p}M$, in such a way that given two vectors x,y∈M, the inner product $G_{p}\left(x,y\right)$ depends smoothly on the point *p*. The tangent space represents the nearest approximation of the manifold by a vector space (Warner, 2013).

3 A Hilbert space ℋ is a complete inner product space, where an inner product is a map 〈⋅,⋅〉:ℋ×ℋ→ℂ , which associates each pair of vectors in the space with a scalar quantity. In particular given x,y,z∈ℋ and a,b∈ℂ

(9) 〈ax+by,z〉=a〈x,z〉+b〈y,z〉,

(10) 〈x,x〉≥0,and〈x,x〉=0⇔x=0,

(11) 〈x,y〉=〈y,x〉.

4 The Dice score over two sets *A* and *B* is defined as $DSC=2\frac{\left|A\cap B\right|}{\left|A|+|B\right|}$.
